# Methods for assessing efficacy of cleaning and disinfection in livestock farms: a narrative review

**DOI:** 10.3389/fvets.2025.1581217

**Published:** 2025-08-18

**Authors:** Iryna Makovska, Evelien Biebaut, Pankaj Dhaka, Leonid Korniienko, Julia Gabrielle Jerab, Laura Courtens, Ilias Chantziaras, Jeroen Dewulf

**Affiliations:** ^1^Veterinary Epidemiology Unit, Faculty of Veterinary Medicine, Ghent University, Merelbeke, Belgium; ^2^Centre for One Health, College of Veterinary Science, Guru Angad Dev Veterinary and Animal Sciences University, Ludhiana, India; ^3^Research Department of Epidemiology and Infectious Diseases, State Scientific Research Institute of Laboratory Diagnostics and Veterinary and Sanitary Expertise, Kyiv, Ukraine

**Keywords:** ATP, ACP, farm hygiene, efficacy of C&D, evaluation of cleaning, evaluation of disinfection, microbiological methods

## Abstract

Cleaning and disinfection (C&D) procedures are essential components of farm biosecurity, aiming to reduce microbial load and eliminate the pathogenic microorganisms in livestock farms facilities. This review examines the various methods used to assess the effectiveness of both cleaning and disinfection, exploring their strengths, limitations, and optimal-use scenarios. For cleaning evaluation, common methods include basic visual inspections, ultraviolet (UV) fluorescence, adenosine triphosphate (ATP) bioluminescence, rapid protein tests (RPT), redox potential, and microbiological swabbing. However, visual inspections and UV fluorescence alone provide only qualitative insights. ATP offers quantitative data, though the accuracy can be influenced by the presence of detergents or disinfectants, requiring careful calibration. Additionally, ATP and RPT testing demands standardization to ensure consistent results. A new promising redox method is fast and more accurate, however still has limited field applicability. Microbiological methods, while highly accurate in detecting microbial contamination, are resource-intensive and therefore not in frequent use for routine evaluation of the cleaning procedures. For assessment of disinfection procedures microbiological tests such as colony-forming unit counts on agar plates, as well as the use of selective media for target microbes or hygiene indicator organisms are more appropriate than non-microbiological tests as they offer direct evidence of microbial elimination. However, these methods can be labor-intensive and time-consuming. Molecular methods can be powerful tools in detecting hard-to-culture organisms, however, are more expensive and require specialized equipment. Given these challenges, our study recommends a comprehensive C&D evaluation protocol, incorporating multiple methods tailored to the farm’s specific biosecurity needs and epidemiological context. This integrated approach improves the reliability and efficiency of C&D monitoring, ensuring robust hygiene management in farm settings.

## 1 Introduction

Hygiene monitoring of farm animal facilities encompasses the observation and evaluation of farm cleanliness. Through this, potential on-farm contamination sources can be identified, and likewise, possible lapses in the implementation of the cleaning and disinfection (C&D) procedures can be identified (1, 2). Inadequate hygiene practices can be associated with various risks and negative outcomes, including disease outbreaks, compromised animal health and welfare, the spread of zoonotic pathogens, economic losses, contamination of animal products, increased antibiotic use, environmental pollution, and finally damage to the industry’s reputation (3–5). Thus, the importance of hygiene management in livestock has been steadily increasing, particularly within the broader context of farm biosecurity (1, 2, 6).

From a biosecurity perspective, proper C&D procedures are important to reduce or eliminate organic and microbial load at the farm level to minimize the infection pressure (3, 7, 8). As was confirmed in previous studies the implementation of proper C&D measures in pig and poultry housing reduced pathogens like *Salmonella* spp. and *Campylobacter* spp. (9–13). Additionally, maintaining adequate hygiene on farms also mitigates the risk of colonization by antibiotic-resistant bacteria (1, 14). At the same time, it was emphasized that inadequate C&D procedures result in residual organic material, which can reduce the effectiveness of disinfectants (15) and create conditions favorable for microbial persistence, including the development of protective matrices that shield pathogens from inactivation (16). Considering that C&D incurs expenses related to working time, purchase of equipment, and consumables, improper C&D can result in a wasteful expenditure of resources (17). That is more concern, that even when C&D procedures are properly implemented, using the correct disinfectant, concentration, contact time, proper temperature, and applied to pre-cleaned surfaces, the elimination of all pathogens cannot be guaranteed (18). This is due to factors such as the formation of biofilms, the development of resistance to cleaning chemicals and/or disinfectants, or the presence of difficult-to-clean or disinfect locations (such as drains and lairage pens’ cracks and holes) (19, 20). Therefore, assessing the efficacy of C&D procedures in livestock facilities is essential to confirm the successful elimination or inactivation of pathogens (21–23).

Generally, checking the efficacy of C&D procedures in animal housing facilities can be carried out as part of routine practices, periodically (scheduled audits), or as emergency response. However, the evaluation of C&D practices on animal farms is often not conducted at the recommended level due to time constraints, limited awareness of available evaluation methods, and insufficient understanding of hidden risks (24, 25). A similar tendency was identified in a study assessing the application of C&D evaluations procedures on pig farms across 10 European countries revealed that only 1% of farms regularly assess the effectiveness of their C&D measures (e.g., with hygienogram) (23). This finding can suggest that farmers, workers, and occasionally veterinarians may lack adequate awareness of proper methods for the evaluation of C&D procedures.

While there are different methods available for evaluation of C&D practices in human hospitals and food industries (17, 26–28), however in farm environments it is still complicated due to several key factors. These include the variability of farm settings, the abundance of organic matter, and the challenges in consistently applying and evaluating the C&D practices across different types of equipment, surfaces, and animal housing areas. Therefore, the aim of the present review is to analyze and evaluate the various methods used to assess the effectiveness of C&D practices/measures and to translate these findings into practical recommendations for animal health professionals /farmers.

## 2 Materials and methods

The literature search for this narrative review was conducted in two rounds, a first one from October 2023 to December 2023 and a second one from May 2024 to June 2024. For both, three primary databases, PubMed, Web of Science, and Scopus, were selected to ensure comprehensive coverage of relevant scientific publications. The search was based on a set of carefully chosen keywords related to the assessment of C&D procedures and farm hygiene, including terms such as: “evaluation of cleaning”; “evaluation of hygiene”; “monitoring of hygiene”; “hygiene monitoring on farms”; “hygiene control”; “hygiene in stables”; “hygiene of surfaces”; “evaluation of disinfection”; “efficacy of disinfection”; “effectiveness of disinfection”; “effectiveness of C&D”; “efficiency of C&D”; “cleaning and disinfection efficacy.”

A thorough evaluation of the selected papers was performed by two co-authors in accordance with the inclusion and exclusion criteria outlined in Table 1.

**Table 1:** The inclusion and exclusion criteria adopted in the literature review process.

	Inclusion criteria	Exclusion criteria
1	Peer-reviewed original research articles, book chapters, and standard guidelines	Manuscripts that were not peer-reviewed, commentary, and conference abstracts
2	Manuscripts written in English	Manuscripts not written in English
3	Studies focusing on pigs, poultry, and cattle farms	Studies focusing on pets, companion animals, wild animals and aquatic species
4	Studies including the effect of C&D on farm surfaces and equipment	Studies including the farm air and water systems

From all relevant publications, a comprehensive review was conducted to extract applicable data on current and potential methods for evaluating the effectiveness of C&D procedures. This systematic analysis yielded key insights into the various approaches/methods used to assess the efficacy of hygiene interventions across different types of farm facilities and production systems. Given the limited number of studies specifically focused on hygiene in animal housing, the review was further expanded to include information from related fields such as the food industry and human medicine as an example of the possibility to expand it to the farm environment. This broader scope provided a more comprehensive understanding of potential methodologies that could be adapted for varied farm conditions. The data extracted were synthesized and presented through a combination of visual representations and detailed tables to enhance clarity and understanding.

### 2.1 Data extraction and synthesis

The selected studies were classified into three main thematic sections based on the type of hygiene monitoring methods used on farms:

(1) Non-microbiological assessment methods including studies focusing on visual, biochemical, and chemical assessment without direct microbial testing.
(2) Microbiological assessment methods including studies with a focus on detection of targeted microbial contamination and/or assessment of hygiene indicator organisms.
(3) Molecular methods including studies describing molecular techniques for the detection and identification of microorganisms.

For each of the identified methods, the strengths, limitations, and practical implications were listed and discussed.

## 3 Results

### 3.1 Overview of C&D procedures in farm facilities

The present review has identified distinct categories of methods used to evaluate the hygiene status in animal housing facilities, whether after cleaning, after disinfection or following the complete C&D process.

An effective C&D regime comprises seven essential steps: (1) Dry cleaning (to remove all organic material); (2) Soaking with a detergent (soaking of all surfaces preferably with detergent for appropriate contact time); (3) Pressure washing (high pressure cleaning with water to remove all dirt); (4) Drying (to avoid dilution of the disinfectant applied in the next step); (5) Disinfection (to achieve a further reduction or elimination of the concentration of the pathogens); (6) Final drying (drying of the stable to assure that animals afterwards cannot come into contact with the residues of used disinfectant); (7) Evaluation (testing of the efficiency of the procedure through sampling of the surface by using applicable methods). The final step (7) should involve testing of the efficiency of the procedure through sampling of the surface by using applicable methods such as visual inspection, ATP testing, or microbial swabbing, etc., to ensure the overall effectiveness of the entire process (8, 18, 29).

### 3.2 Non-microbiological assessment methods

#### 3.2.1 Visual assessment

##### 3.2.1.1 Basic visual inspection

Visual inspection is a common and conventional method for assessing hygiene in animal facilities, offering a quick evaluation of the cleaning effectiveness of stables, equipment, and materials, without requiring specialized tools (30). It can be applied both before and after cleaning. Performing it after dry cleaning but before detergent cleaning or disinfection helps addressing gaps and may reduce residual organic material.

###### Description of the test

This approach is widely used, but only few studies described in detail the procedure. In a poultry farm study, the inspection was conducted for equipment and buildings after cleaning and before disinfection. Each building was divided into four sections identifying the specific control points for assessment based on the grid approach described by Rose et al. (31), and calculated cumulative scores as percentages, with 100% indicating perfect cleanliness (32). In pig farms, a three-point grading system was applied to evaluate visible soiling: 1 = satisfactory (no visible soiling), 2 = sufficient (minor soiling), and 3 = unsatisfactory (visible soiling) (24). Similarly, a poultry farm study adopted a three-tier scale: 0 = soiled, 1 = partially cleaned, and 2 = clean (20). However, as was highlighted by Heinemann (33), the lack of standardized definitions for “clean” and “dirty” makes such assessments subjective. Visual hygiene assessments of milking and feeding equipment in cattle farms have been employed in multiple studies using standardized scoring systems based on the presence of visible organic residues. A commonly adopted 4-point scale evaluates equipment surfaces that come into contact with colostrum or milk, where: score 1 denotes visibly clean equipment with no detectable fecal, milk, or colostrum residues; score 2 indicates minimal residual traces; score 3 reflects clearly visible contamination; and score 4 represents extensive contamination, often involving manure, milk, or colostrum deposits (34, 35). In some studies, visual scoring charts were used for evaluating items such as buckets and nipples (36), with additional modifications for esophageal and automatic milk feeders, taking into account factors like tube transparency to assess internal cleanliness. All assessments were performed by a single evaluator to ensure consistency (34). The goal should always be to achieve the better possible visual hygiene score immediately after the cleaning process and just before the equipment is used again (37).

###### Advantages

Visual inspection is a quick and simple method to do the checking, allowing evaluators (staff, auditors, and other stakeholders) to spot visible dirt or residue efficiently. If a surface fails this test, the use of more advanced methods like rapid tests, microbiological analyses, or even proceeding with disinfection is unnecessary (8, 18).

###### Limitations

The main limitations of basic visual inspection are its subjectivity, reliance on the inspector’s perception, and external factors such as lighting and surface color (24). Only visible areas can be assessed, potentially missing hidden spots. For example, a poultry farm study found high adenosine triphosphate values on drinking cups, drain holes, and floor cracks after cleaning, which confirmed that these areas still contained notable amounts of organic material and/or bacteria after cleaning despite visual inspections showing cleanliness for the last two (20). Similar findings from studies on colostrum-feeding equipment in cattle farms revealed that nipples appearing clean upon visual inspection were found to be contaminated when assessed using luminometry, likely due to hard-to-see internal surfaces, especially in dark or narrow designs (34).

###### Overall reflection

Visual assessment is generally considered a poor indicator of cleanliness on farm (34–36). Although it is highly subjective (38) and only offers a qualitative assessment, making it difficult to track improvements, it remains a valuable and integral tool within a comprehensive C&D evaluation protocol (39, 40). To improve reliability, the visual inspection process on farms should be standardized by defining clear criteria (8, 31), training and calibrating inspectors regularly (37, 38), using detailed scoring systems and checklists (32), and incorporating digital imaging or AI tools (41) for more objective assessment. Emerging AI tools, such as computer vision and machine learning algorithms, can enhance the objectivity of farm visual inspections by automatically detecting hygiene deficiencies and standardizing image-based evaluations.

##### 3.2.1.2 Ultraviolet (UV) fluorescence

UV fluorescent markers, also known as UV tracers or UV fluorescent dyes, provide a visual indication of areas in animal houses that are not properly cleaned (42).

###### Description of the test

**Figure 1:**
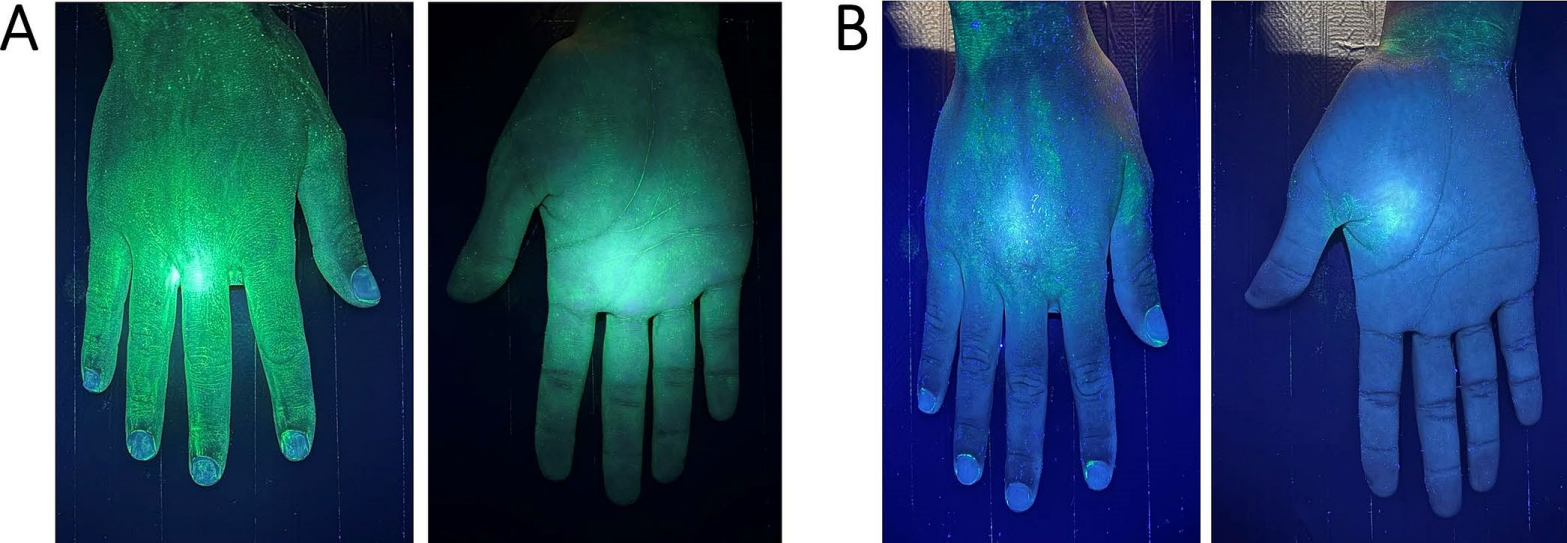
Hand examination under UV light in a box: **(A)**—pre-cleaning view (after applying UV gel the fluorescent light marks the whole hand) and **(B)**—post-cleaning view (fluorescent spots mark the areas that are not properly cleaned).

UV fluorescent markers emit visible fluorescence under UV light and are easily removed by wet mopping. In animal housing facilities, these dye-based markers are typically applied to surfaces prior to cleaning, and the cleaning process is considered effective if more than 90% of the marker is visibly removed. In studies of veterinary and hospital cleanliness, outcomes are categorized as “clean” (mark faded) or “dirty” (mark persists) (Figure 1) (42, 43). UV markers are also used to assess hand hygiene in hospitals using UV light boxes (44), with a 4-point scale from “very dirty” to “very clean” (45).

An American study on pig farms highlighted the broad application of specialized fluorescent gels and powders, such as “Glo Germ”, which can simulate germs or contaminants visible under UV light. These tools have demonstrated effectiveness as educational aids, improving biosecurity practices across various contexts. They are particularly valuable for training individuals in identifying contamination risks, serving as practical and engaging tools in swine facilities and other agricultural settings (46).

In addition, a study conducted in the USA applied deep learning algorithms, Xception and DeepLabv3+, to analyze images of surfaces and equipment in the food industry. The models accurately distinguished between contaminated and clean surfaces with 98.78% accuracy (41). This approach holds potential for future application in farm facilities.

####### Advantages

UV fluorescent markers are relatively easy to use and have low costs related to them (47). They do not require specialized equipment or complex procedures, making them accessible and practical for routine assessments. UV fluorescent markers are non-destructive to surfaces and most of them are non-toxic, posing minimal risk to humans or animals (41).

####### Limitations

Major limitations are additional work/time for marking surfaces prior to cleaning procedures and that this methodology requires a UV light source (42). Its effectiveness depends on the observer’s ability to detect fluorescence, which can be influenced by lighting conditions and improper use of UV light. Additionally, it does not provide microbial contamination data.

####### Overall reflection

UV fluorescence can be useful for assessing the efficacy of cleaning procedures. They are especially used as a training and educational tool to raise awareness and promote better cleaning as well as hand hygiene practices (44, 45). Works best when subjectivity is minimised using pre-printed scoring templates (such as % of marker removed) or pictures.

##### 3.2.1.3 Adhesive tape evaluation

Adhesive tape sampling methods have been used in clinical, environmental, and food microbiology since the early 1950s (48). This sampling method can be used for porous surfaces or uneven areas that may be difficult to inspect visually directly or to sample using swabs or agar contact plates (49).

###### Description of the test

The test methodology involves pressing adhesive tape onto a surface to collect residual particles, both organic and inorganic, which are subsequently visually inspected or microbiologically cultured to evaluate C&D efficiency (48). Advanced techniques like spectroscopy or microscopy may be used for further analysis, helping to identify specific particles or microorganisms.

###### Advantages

The method is simple, cost-effective, and requires minimal equipment, making it suitable for routine use. It is versatile, easily covering irregular surfaces and hard-to-reach areas. The collected samples are manageable, transportable, and non-destructive (50). This method furnishes a qualitative appraisal of the presence of visible residues, aiding in the identification of areas necessitating further attention.

###### Limitations

The use of adhesive tape sampling is limited to collecting contaminants from small surface areas and does not capture those embedded in deeper layers of rough surfaces (e.g., concrete). Its effectiveness is influenced by factors such as the sampling technique, applied pressure, and the type of tape used. Additionally, it may miss larger or non-adherent contaminants, leading to potential inconsistencies in results. If only visual analysis is done, interpretations can be subjective.

###### Overall reflection

The technique is simple and requires minimal equipment, making it an accessible option for routine monitoring of small surface areas in animal farm environments It is primarily suited for detecting visible residues rather than accurately quantifying the microbial load on surfaces.

#### 3.2.2 Biochemical assessment methods

##### 3.2.2.1 Adenosine triphosphate (ATP) bioluminescence technology

ATP analysis is a quantitative method that can be used for monitoring hygiene after C&D (8, 20). It is typically applied to high-touch surfaces and critical control points, focusing on areas prone to contamination.

Traditionally, this technique is more commonly implemented in human medicine and the food industry to assess potential contamination points as well as C&D regimen. However, recent studies have demonstrated that this method has gained popularity and is increasingly utilized to assess hygiene effectiveness in veterinary medicine, especially for the evaluation of specific surfaces in scientific studies (38, 39, 51). For example, in dairy farms, studies highlighted the potential of ATP as an on-farm tool for evaluating the hygiene of rubber liners (35), for cleanliness of equipment used to collect and feed colostrum (36, 52), for assessing the cleanliness of feeding equipment in pre-weaning calves (34), and for cleanliness of milking equipment (53). In the poultry industry, ATP bioluminescence was used to assess cleanliness in broiler houses from 12 different sample points (such as floor, wall, drinking cup, feed hopper, loose material, etc.) (20), in battery cages and on-floor layer houses (32), in carcass processing environments to identify critical control points and equipment surfaces (54). In pig farming, ATP assay was used for testing floor corners, floor centers, and feeding troughs in an empty pig farrowing unit before and after standard cleaning procedures (55).

###### Description of the test

ATP analysis detects biological residues, including cells from plants, animals, and microorganisms, as ATP is the universal energy source in living cells (17). As the dirt that is left in farm animal housing is often a mixture of feces, urine, leftover feed, animal cells, and bacteria, the ATP levels provide an indication of surface cleanliness (33).

**Figure 2:**
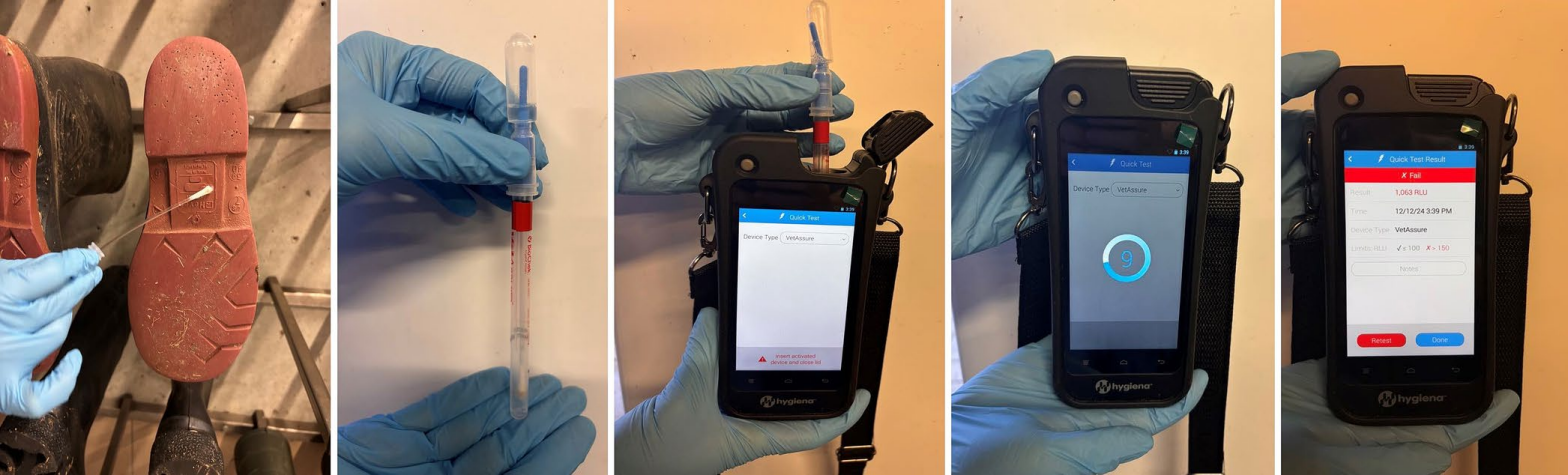
ATP procedures: measuring surface contamination levels (swabbing the boots after cleaning; mixing with reactive solution; placing the vial into ATP testing device; waiting for results 10 s; receiving the results).

ATP analysis operates on the principle of introducing a solution containing a lysis reagent, luciferin substrate, and luciferase enzyme to a swab sample. The lysis reagent facilitates the release of ATP from all living cells. When ATP is released, it is utilized by the luciferase enzyme to convert the luciferin substrate, resulting in a bioluminescent reaction that produces light. The intensity of this light correlates with ATP levels (8). The light is measured in relative light units (RLUs), where higher RLUs indicate greater contamination (Figure 2). ATP levels vary by cell type (e.g., yeast, bacteria) and growth phase, but regulatory mechanisms maintain a consistent ATP pool (17). Thus, routine cleaning assessments should be compared with baseline data of acceptable cleanliness values. ATP bioluminescence thresholds for hygiene assessment vary considerably across studies, depending on animal species, facility type, and the specific surface or equipment being tested. For instance, Lindell et al. (35) reported ATP cutoffs for milking equipment ranging from ≤150 relative light units (RLU) to indicate cleanliness, to ≥300 RLU as indicative of contamination. In the context of colostrum feeding equipment, Buczinski et al. (52) proposed a general threshold of <1,000 RLU for clean devices, and ≥1,000 RLU for contaminated ones. Similarly, Van Driessche et al. (38) classified ATP readings as follows: ≤500 RLU (clean), 501–1,000 RLU (alert), and >1,000 RLU (fail). A wider range was observed by Vilar et al. (53), who reported thresholds from <152 to <1,1824 RLU, depending on sampling sites within cattle operations. In poultry barns, Mateus-Vargas et al. (56) considered values ≤150 RLU as indicative of effective cleaning, with readings above this level reflecting insufficient hygiene. For pig fattening units, Heinemann et al. (33) identified >500 RLU as a threshold indicating inadequate cleanliness of pen surfaces. These variations underscore the importance of establishing facility-specific pass/fail benchmarks when using ATP monitoring as a routine hygiene assessment tool, as recommended by Heinemann et al. (24).

###### Advantages

ATP bioluminescence tests swiftly detect organic residues, providing quantitative results within a short time (around 5–10 s) without the need for microbial cultivation. This allows for immediate feedback on surface cleanliness, enabling real-time adjustments and interventions.

###### Limitations

A major limitation is the absence of specific standards for the RLUs to define cleanliness (57). Each ATP device manufacturer employs its own RLU scale, based on their specific ATP luminescence curve, making all measurements and RLU values relative to that particular system. Furthermore, users should understand that the ATP value does not relate directly to the microbial load of surfaces, even when only bacteria are measured. Similarly, non-microbial sources of ATP, such as plant material, could lead to an overestimation of microbial contamination (33). Therefore, if ATP values are very high, additional microbial testing is recommended for verification. Although the ATP assay can lead to a false positive result, the cost of an additional cleaning is typically lower than the potential expenses associated with an infectious disease outbreak, such as PEDV (Porcine Epidemic Diarrhea Virus) or PRRSV (Porcine Reproductive and Respiratory Syndrome Virus) (39, 58).

Another limitation of the methodology is that residual detergents and disinfectants may interfere with the ATP bioluminescence reaction and alter RLU values leading to inaccurate results (27, 59, 60). As was observed in previous studies, among the nine chemicals that were tested for impact on ATP, quaternary ammonium was the only one that increased log_10_ RLU measurements. In contrast, hydrogen peroxide and peroxyacetic acid sanitizer caused larger log_10_ RLU reductions in ATP measurements from organic sources (chicken exudate) compared to pure ATP sources (61). For this reason, it is advisable to use commercial bioluminescence detection kits that include neutralizers to mitigate the effects of detergents and sanitizers (61) or leave a drying period of a minimum of 12 h after disinfection of a surface before using ATP to evaluate cleanliness. In addition, it’s important to note that ATP systems are also unable to detect spores, viruses, or prions, as they do not contain ATP (61). Moreover, when samples are collected from areas that are not visibly clean, the contamination load of the swab may be too high, preventing an accurate ATP measurement, which could result in false negative results. Thus, it is recommended to use this technique exclusively on visually clean surfaces, as advised by the manufacturers (52). A similar statement was confirmed in a study in pig barns which found a strong correlation (r = 0.698, *p* < 0.001) between ATP and total aerobic bacteria (TAB) levels after cleaning. However, the correlation was weakened in the presence of fecal bacteria, emphasizing the need for thorough cleaning and visual inspection before testing (55).

In comparison to the food industry, where ATP testing is already standardized, its application for veterinary purposes still requires further standardization. Until this is achieved, each manufacturer should, at the very least, provide its own upper and lower RLU limits for application within the veterinary context (55). To accurately interpret the hygiene status while using ATP testing on a regular basis, the user must set a pass or fail benchmark as was suggested by Heinemann et al. (24).

###### Overall reflection

Despite its limitations, ATP bioluminescence is increasingly popular (especially in the frame of scientific projects) for environmental cleanliness monitoring in farm facilities serving as a technique suitable for real-time assessment of surfaces by focusing on dirt absence, not microbial count where cleanliness, but not sterility, is required (20, 35, 57). However, opinions on the effectiveness of ATP testing vary. Some argue that ATP testing should not replace quantitative methods for determining microbial load and should, therefore, be supplemented with microbiological methods (35, 57, 58, 62–64). On the contrary, other researchers have confirmed that ATP testing can be a cost-effective alternative to microbiological methods along with visual inspection (55) and alone (24).

However, based on the analysis of the different studies there are important prerequisites: in case of use after cleaning, the ATP testing should be used only on visually clean surfaces, and in case of using ATP testing after disinfection, it can only be applied when surfaces are completely dry, in both cases in order to avoid false results. A future standard approach requires identifying a benchmark and establishing a cut-off value to alert farmers when extra cleaning is needed (65).

##### 3.2.2.2 A3 system (ATP+ADP+AMP)

The A3 test quantifies the total adenylate content, ATP, ADP (adenosine diphosphate), and AMP (adenosine monophosphate), collectively on surfaces to assess cleanliness. Therefore, measuring total adenylates (ATP + ADP + AMP, known as A3) may provide a more reliable indicator of residual contamination that can lead to biofilm formation and other contamination (65).

###### Description of the test

In this method, samples collected from surfaces are processed to convert all adenylates into ATP using enzymatic reactions. The resulting ATP is then measured using a standard bioluminescence reaction catalyzed by the luciferase enzyme. Because the test captures all forms of adenylates rather than ATP alone, it provides a more accurate measure of residual organic matter, including degraded biological material, on cleaned surfaces.

###### Advantages

By measuring all three adenylates (ATP, ADP, AMP), the test captures residual organic matter even when ATP has partially degraded, increasing detection accuracy. A3 levels correlate with residues that promote biofilm formation, enabling earlier identification of sanitation issues. Like ATP bioluminescence, A3 testing can be performed quickly with portable luminometers (66).

###### Limitations

A3 testing equipment and reagents are less common and can be more expensive than standard ATP tests. The inclusion of ADP and AMP can complicate result interpretation, requiring calibration and training. Some cleaning agents or sample matrices may affect adenylate stability or detection, causing false readings (67).

###### Overall reflection

The A3 test offers a more comprehensive and reliable alternative to traditional ATP testing by accounting for ATP degradation products, improving cleanliness assessments in environments where heat or chemicals degrade ATP. Finally, this approach allows detection of degraded organic residues that may not contain intact ATP but still pose hygiene risks, providing a more reliable indicator of surface contamination. Although promising, its broader adoption depends on further validation, cost reduction, and user training to interpret results accurately. Integrating A3 testing with other hygiene monitoring methods can enhance overall sanitation control.

##### 3.2.2.3 Luciferase-based methods

All ATP bioluminescence tests use luciferase, but not all luciferase-based tests are ATP tests. Some are designed to find live pathogens. These tests are more specific than general ATP tests and can confirm if harmful microbes (such as *Salmonella*, *Listeria*, or *E. coli*) are still present after C&D procedures.

###### Description of the test

Luciferase-based microbe detection combines the bioluminescent reaction of the luciferase enzyme with biological targeting mechanisms, such as bacteriophage-based systems or pathogen-specific genetic probes. Phage-based tests use luciferase as a biosensor to detect specific microbe, often via genetically modified bacteriophages or molecular probes that only activate the luciferase signal when a viable target pathogen is present (68, 69). Genetic probe-based methods use nucleic acid amplification coupled with luciferase to quantify specific bacterial DNA (70). These approaches ensure that luminescence occurs exclusively in response to viable and correctly identified microbe, enhancing both specificity and reliability.

###### Advantages

Offers high specificity, as it targets viable pathogens rather than general organic material, allowing for accurate confirmation of disinfection effectiveness. The test delivers results within hours, significantly faster than traditional culture methods, and is sensitive enough to detect low pathogen levels. It is also non-destructive, requiring minimal sample preparation, and the intensity of luminescence correlates quantitatively with pathogen concentration (68, 71).

###### Limitations

More complex, time consuming and expensive than ATP tests. Require specific reagents and instruments, require skilled lab personnel to carry them out, typically suited for lab or semi-lab settings, which limit field applicability. The test generally targets specific pathogens, so it is not suitable for broad-spectrum screening. Additionally, sample matrices can sometimes interfere with the luciferase reaction, causing false results. Proper sample collection and handling, as well as operator training, are critical to ensure accuracy (68, 71).

###### Overall reflection

Luciferase-based pathogen detection provides a valuable balance between rapid turnaround and specificity for viable pathogens, making it a useful tool for verifying the effectiveness of C&D protocols on farms. However, it is best employed as part of an integrated hygiene monitoring strategy that also includes complementary methods such as ATP bioluminescence and microbiological cultures to ensure comprehensive pathogen detection and control (68).

##### 3.2.2.4 Rapid protein tests (RPT)

RPTs are widely used across multiple industries, including the food industry, healthcare, and environmental monitoring. On farms, rapid protein tests can be applied to assess the cleanliness and hygiene of equipment, surfaces, and animal housing facilities. By detecting protein residues, these tests can reveal the presence of organic contaminants, such as manure, feed residues, or milk, which may potentially carry harmful microorganisms (24).

###### Description of the test

Rapid protein tests detect protein residues through chemical reactions that cause a color change, typically within 1–15 min. Using swabs, test strips, or pads, samples are mixed with a reagent to induce a color shift, which indicates the presence and extent of protein contamination. The color change is often assessed using a predefined scale, such as the 5-point scale used in a German study to evaluate the intensity of the shift from green to violet after 15 min (24). The semi-quantitative scoring system typically ranges from 1 (no detectable color change) to 5 (intense color change), with scores exceeding 2 or 3 generally interpreted as indicative of insufficient cleaning or inadequate hygiene.

###### Advantages

RPTs allow for the immediate semi-quantitative assessment of surface cleanliness. According to Heinemann et al. (24), rapid protein tests are highly inviting for on-farm monitoring due to their short duration, in contrast to microbiological techniques. While the interpretation of color changes could potentially be subjective, this can be supplemented by inexpensive tools to measure and/or record results when needed (24).

###### Limitations

These tests do not detect non-organic substances, chemical or residues (24, 33, 53, 72). Their sensitivity can vary across surfaces and test brands, leading to inconsistent results that require validation and standardization. Additionally, detergent or disinfectant residues may interfere with test outcomes, causing false positives or negatives. To address this, low-residue disinfectants or post-application residue removal methods can be used, although the term “low residue” does not guarantee zero residues, and levels are typically defined as <25 ppm (24).

###### Overall reflection

Results of rapid protein tests are often qualitative (pass/fail) or semi-quantitative, providing less precise information. When comparing ATP and RPT, ATP tests more accurately reflect subtle differences, whereas rapid protein tests only allow for the visual recognition of coarse color graduations (24).

#### 3.2.3 Chemical assessment method

##### 3.2.3.1 Redox potential measurement

The redox potential method is one of the complex indicators of the physiological state of microbial cultures and its measurement could be a useful tool for the qualitative and quantitative determination of microbial contamination (73).

###### Description of the test

The redox potential method relies on oxidation–reduction reactions in biological systems, driven by microbial activity. During microbial growth, biological oxidation leads to oxygen depletion and the production of reducing compounds, causing a measurable decrease in the redox potential (Eh) of the medium (74). This change, governed by the Nernst equation, serves as an indicator of microbial activity and contamination levels (73). Researchers have validated this approach for coliform bacteria, finding a strict linear correlation between termed time-to-detection and the log of the initial CFU count (73, 75). Where the ‘time-to-detection’ is the interval between inoculating the sample into the redox-sensitive medium and the moment when the measured redox potential (Eh) drops to the chosen cutoff, indicating significant microbial oxidation and reduction activity.

###### Advantages

Redox potential measurement can detect microbial activity within 16 h, faster than the minimum 24-h incubation required for the reference plate culture method. This technique is efficient and can be tailored to specific bacterial strains by using selective media, as each bacterial strain exhibits a unique kinetic pattern in terms of redox potential change. This makes the method not only faster but also adaptable to different types of microbial contamination (75). Recent studies have led to the development of mobile devices for measuring microbiological activity on farms (still in progress), utilizing the redox potential measurement technique, opening new ways to its use by the sector. Also, mathematical models have been successfully used to describe the specific shape of redox potential curves for several bacteria optimizing the readings and leading to higher accuracy when classifying bacterial species (75).

###### Limitations

The redox potential is a complex indicator influenced by various factors, requiring careful interpretation. Automation is possible but demands advanced redox electrodes and measurement systems. Despite its potential, the method has historically seen limited application (73, 75, 76). Due to the non-availability of devices for field conditions (available devices are only non-portative), this method needs more improvement.

###### Overall reflection

This method can be a cost-effective alternative for monitoring microbial contamination. Its adaptability and minimal environmental control requirements make it a valuable tool, though broader adoption and technological improvements are necessary to fully leverage its capabilities (73, 75). Therefore, integrating redox potential monitoring, based on microbial metabolic activity reducing oxidation–reduction potential, can provide faster, indirect microbial detection and offers potential for field-adapted C&D evaluation.

#### 3.3 Microbiological assessment methods

Microbiological assessment is critical for detecting and measuring microorganisms on surfaces, encompassing a wide range of microorganism types (55). Unlike non-microbiological testing, microbiological ones may provide quantitative data on contamination levels through viable colony counts (77) offering insight into the efficacy of C&D (78).

Culturing techniques form the cornerstone of microbiological assessments, enabling the growth and enumeration of microorganisms from collected samples. The mesophilic aerobic total viable count (TVC) is a widely used parameter for assessing surface cleanliness, reflecting the presence of a total number of aerobic and facultative anaerobic microorganisms. Additionally, indicator microbes like *Enterobacteriaceae*, *E. coli*, and total coliforms are used to assess faecal contamination, aiding in identifying hygiene lapses (20, 22, 33). Specific pathogens such as *Salmonella* spp., *Campylobacter* spp., and methicillin-resistant *Staphylococcus aureus* (MRSA) are also commonly targeted in assessments (18, 33, 79).

Microbiological assessment involves collecting microorganisms from surfaces using various techniques, including swabbing and agar contact plates (ACP) (17). A brief description of these two commonly used techniques is provided below.

##### 3.3.1 Swabbing

Swabbing is a versatile sampling technique that involves wiping targeted surfaces with swabs made of materials like cotton, rayon, or nylon, which are then processed for microbiological analysis.

###### Description of technique

**Figure 3:**
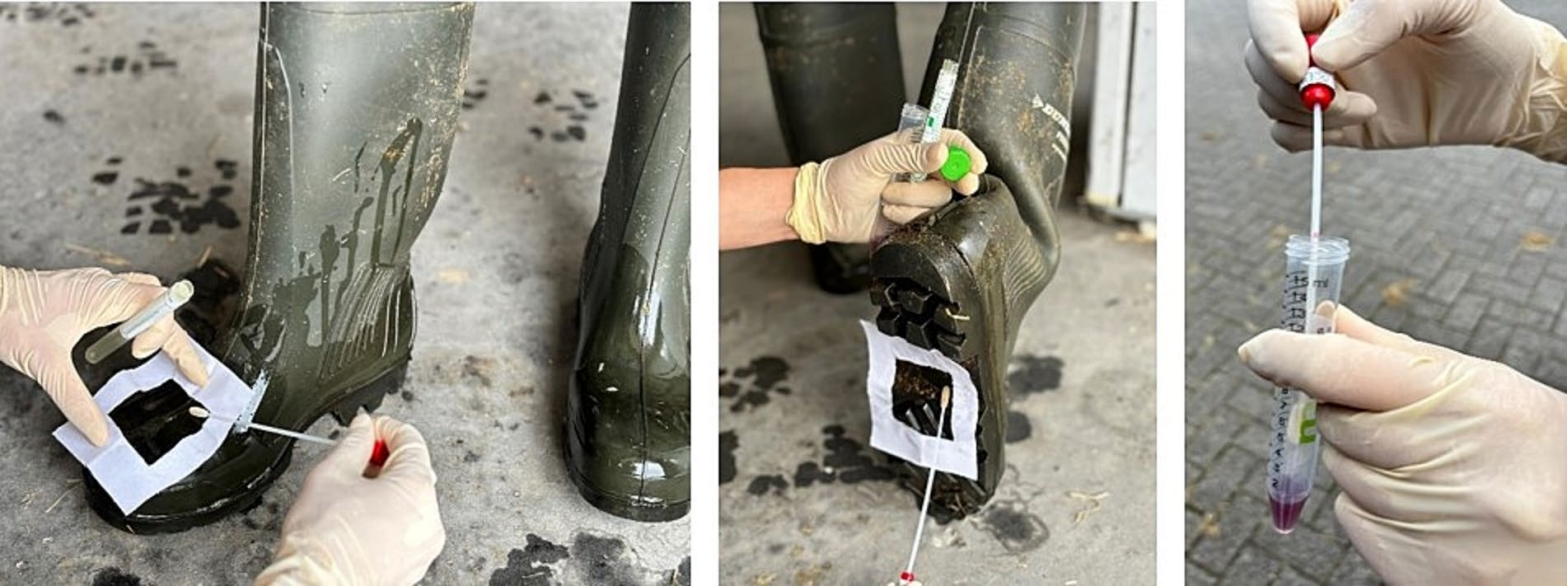
Boot swab sampling process: a swab is wiped horizontally and vertically with rotation over a marked area and placed in a transport media for further assessment.

For direct swabbing, a sterile frame marks the sample area, and a dry or pre-moistened swab is wiped horizontally and vertically with rotation. The swab is then placed in a transport media for microbiological analysis (Figure 3).

**Figure 4:**
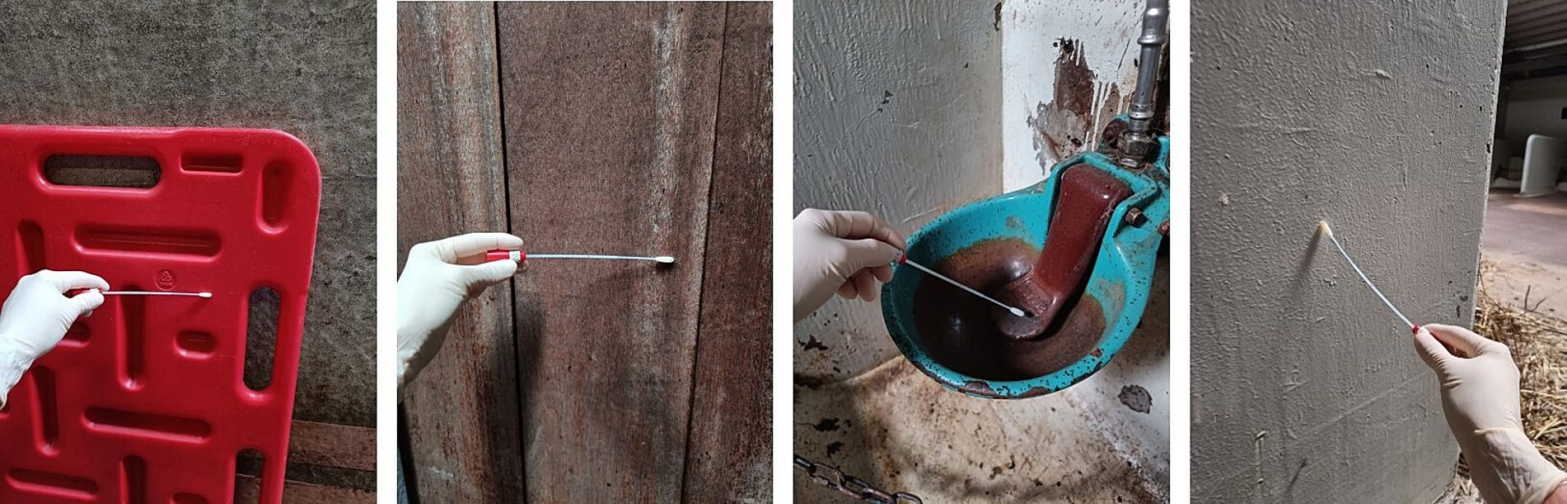
Swabbing of equipment, drink pillars, and walls in farm environments.

This technique is highly effective for sampling irregular or hard-to-reach areas, such as artificial teats or inside equipment (53). Swabbing is commonly used to sample the animal farms from the floor, wall, drinkers, and feeders (Figure 4). The boot sock or boot swab sample method is recommended to examine *Salmonella* spp. occurrence in poultry houses (CR (EU) No 200/2010) (24). This technique also involves pulling sterile disposable hairnets or cotton covers over disinfected shoes (“sock” samples) and walking a defined number of steps through the barn. This method was previously used to detect *Campylobacter* spp., MRSA, total aerobic bacteria, and fecal indicator bacteria such as Enterococci and Enterobacteriaceae (33, 79). In the study by Mateus-Vargas et al. (56), boot swab samples collected by walking over poultry barn floors were used to evaluate C&D efficacy through log₁₀ reductions in total aerobic counts (TAC). The results demonstrated a 3 log₁₀ reduction in TAC following cleaning and a reduction of less than 2.5 log₁₀ after disinfection. Similarly Luyckx et al. (20, 22), reported a decrease in total aerobic flora from 7.7 ± 1.4 to 4.2 ± 1.6 log CFU/625 cm^2^ after disinfection using swab-based culture methods in poultry barns. Although no definitive threshold was proposed, lower bacterial counts were consistently associated with improved hygiene status. In cattle farm settings, Lindell et al. (35) employed swab sampling followed by culture for TAC assessment, where post-cleaning levels below 2–3 log CFU/cm^2^ were generally considered indicative of acceptable surface cleanliness.

###### Advantages

Swabs can be further processed to quantify total microbial contamination, detect specific pathogens, or identify indicator organisms. The type of swab and the choice of moistening and transport solution can significantly impact the recovery of microorganisms, with studies showing nylon flocked swabs with non-growth-enhancing moistening solutions as particularly effective for wet surfaces (28). In cases where swabs are taken after disinfection, the use of a neutralising transport medium ensures that residual disinfectants in the sample are inactivated, enabling an accurate bacterial count during processing. A key advantage of swabs is the ability to dilute the transport solution, which helps prevent microbial overgrowth, an option not available with ACP. Additionally, unlike ACPs, swabs allow the same sample to be plated onto multiple types of agar, enabling the detection and enumeration of different bacterial species from the exact same swabbed surface.

###### Limitations

Swabs as a sampling method have some limitations, including being labor-intensive and highly susceptible to variability in sampling technique. The process is time-consuming, detects only culturable bacteria, and requires access to laboratory facilities. Additionally, there is no universally accepted cut-off for interpretation, and results typically take 2–3 days to obtain (8, 17, 56).

###### Overall reflection

Swabs are useful for certain surfaces that are challenging to sample and provide evidence-based feedback on the effectiveness of C&D protocols.

##### 3.3.2 Agar contact plating (ACP) method

ACP is another essential tool for microbial sampling, particularly for smooth, dry surfaces. In agricultural settings, such samples are frequently taken as part of evaluations after production cycles to evaluate the effectiveness of C&D protocols. Additionally, regulatory bodies may implement ACP testing if specific pathogens are suspected on a farm.

###### Description of the methodology

**Figure 5:**
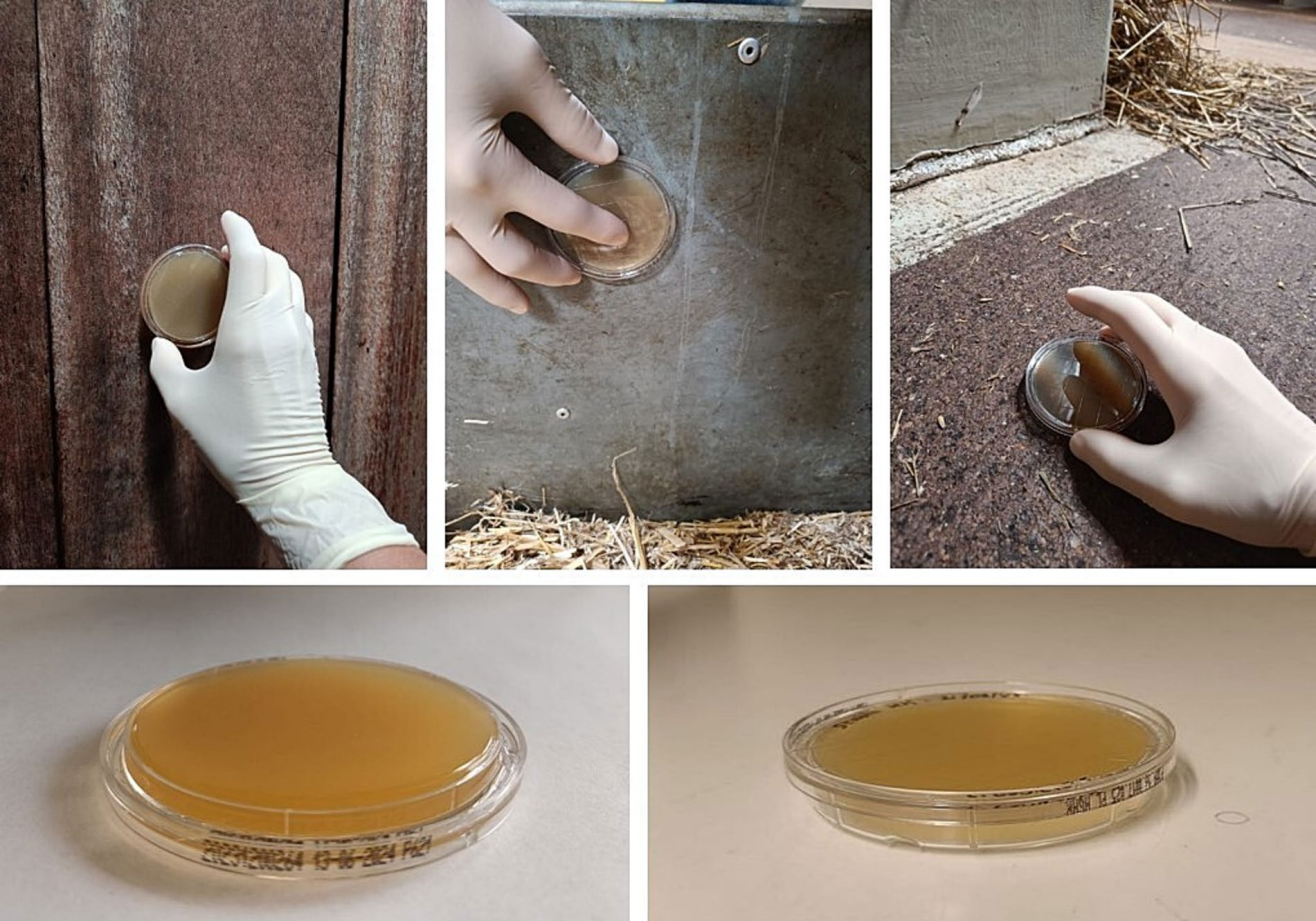
Sampling stables, walls, and floors by using agar contact plating.

The choice of culture medium depends on the targeted bacterial species (33). ACP involves pressing (for several seconds) convex agar plates onto surfaces to capture microbial contaminants (Figure 5).

Further, these plates are incubated to allow bacterial colonies to grow, which are then counted to estimate microbial contamination levels. ACP results are typically expressed in CFU (Colony-Forming Units) or TAC (Total Aerobic Count), and these metrices are commonly referred to as a hygienogram. The CFU count reflects viable, colony-forming microorganisms, indicating microbial activity, while TAC measures all aerobic microorganisms present on a non-selective agar plate. The low CFU/TAC indicating the good hygiene and high CFU/TAC pointing to inadequate cleaning or contamination. The application of ACP for hygiene monitoring has been described in previous studies, where results are translated into a hygienogram scoring system that assigns values from 0 (very good disinfection) to 5 (very poor disinfection) based on colony counts (20). According to Mateus-Vargas et al. (56), this scoring system allows for the categorization of C&D outcomes as: “good” (score ≤ 1.5), “satisfactory” (score 1.6–2.9), and “poor” (score ≥ 3.0). In practical applications, Huneau-Salaün et al. (32) reported that, for poultry farms, total aerobic counts below 200 CFU/25 cm^2^ are considered acceptable, while higher counts reflect microbial overgrowth and inadequate hygiene. On pig farms, the upper threshold for acceptable contamination has been set at 33.3 CFU/cm^2^ (33).

###### Advantages

ACP provides standardized sampling of smooth surfaces with minimal processing and allows for direct colony enumeration, making them particularly suitable for use in hygienograms (20, 33). Compared to swab sampling, ACP offers a simpler and less labor-intensive alternative by enabling direct contact with surfaces, thereby eliminating the need for additional tools such as transport media or extra steps like enrichment and plating. Typically, ACPs use basal agar media, which support the growth of a broad spectrum of microorganisms. However, the agar plates can be customized by using selective media to target specific bacterial types. ACP is particularly advantageous for providing quantitative data about the microbiological load and tracking contamination trends.

###### Limitations

ACPs are limited to flat, smooth surfaces and cannot be used effectively on curved, uneven, or porous surfaces. They may also underestimate contamination from biofilm-embedded microorganisms and are prone to colony overgrowth if the sampled surface is heavily contaminated or not properly cleaned beforehand (8, 24). Like other microbiological assays, ACP demands an incubation period, ranging from hours to days, depending on species and conditions. This might not suit real-time result needs. Also, the interpretation and counting of the colonies on ACP might be subjected to observer variation due to variability in colony size, colour, and morphology (24).

###### Overall reflection

Agar contact plates are a practical sampling method for evaluating C&D in animal farms, making them a reliable method for surface contamination checks.

##### 3.3.3 Targeting specific pathogens

**Figure 6:**
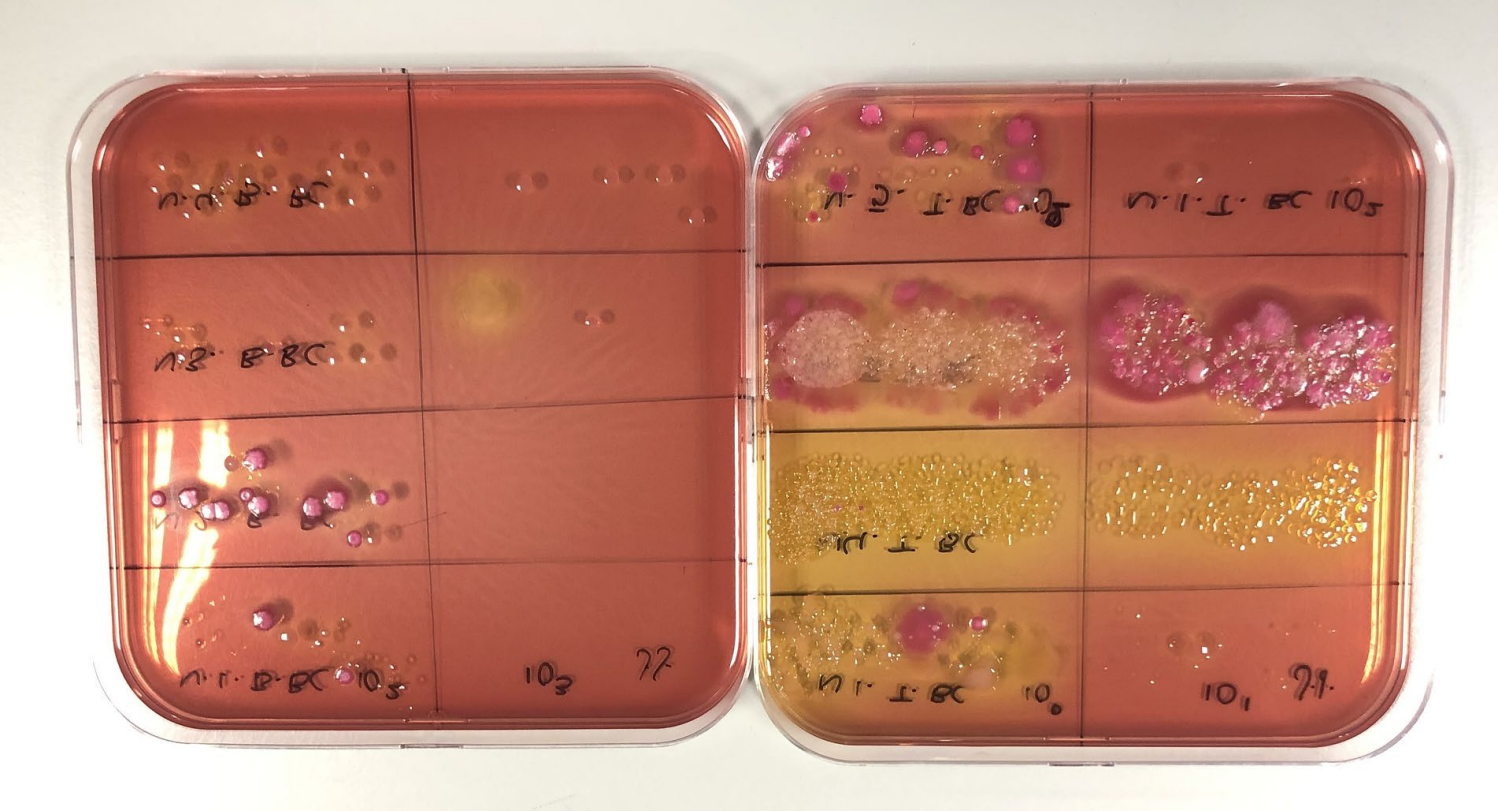
Use of selective MacConkey agar for detecting Gram-negative bacteria and differentiating based on lactose metabolism (pink colonies are lactose fermenting, while non-pink colonies are non-lactose fermenting).

Both swabs and ACP can be applied for selective plating techniques, which enable the identification and quantification of specific microorganisms (80). Selective media, such as Eosin Methylene Blue agar for *E. coli*, Xylose Lysine Deoxycholate agar for *Salmonella* spp., and Campylobacter Blood-Free Selective Medium for *Campylobacter* spp., are commonly used for targeting pathogens of interest (33, 81). These selective media are particularly valuable during outbreaks or persistent health issues, as they allow for precise pathogen detection and quantification (82, 83). Microbiological plating focuses only on viable cells, allowing selective plating techniques to provide a more accurate assessment of disinfection efficacy by confirming the survival or elimination of targeted pathogens (Figure 6).

However, while selective plating is effective in identifying specific bacteria, it may not capture the full spectrum of microbial diversity present on surfaces, potentially overlooking non-target organisms (84). Nevertheless, these approaches remain essential for evidence-based evaluation of C&D protocols or during investigation of the disease outbreaks due to suspected pathogen(s).

The use of indicator organisms such as *Enterobacteriaceae*, *E. coli*, and *Enterococcus* spp. provides valuable information about the overall hygiene status of a facility (17, 20). In general, criteria for hygiene indicators include being more abundant than the pathogen to enhance detection (85), having a survival rate similar to or greater than the pathogen in the environment (24, 33) and being easily detectable with reliable, faster, and safer methods than those used for the pathogen (86). For example, *E. coli* serves as an indicator of fecal contamination and correlates with the likelihood of detecting other enteric pathogens. Past studies have shown that *E. coli* can be a suitable index organism for detecting the possible presence of *Salmonella* spp. (85). Further Luyckx et al. (20), observed that *Enterococcus* spp., due to their resilience, persistence on surfaces, and higher probability of recovering, may be even more effective indicators of cleaning efficacy compared to *E. coli*. The choice of hygiene indicators depends on the farm’s specific needs, the type of animals, and potential pathogens of concern. Since current hygiene indicators primarily focus on fecal contamination; the ideal goal is to develop hygiene indicators specifically designed to address biosecurity lapses in different farm areas.

### 3.4 Molecular assessment methods

Molecular methods are particularly advantageous for their potential to identify microbial species strains, and even discrete subtypes, including pathogenic variants, with enhanced sensitivity and specificity (33). Molecular tools like traditional polymerase chain reaction (PCR) and real-time PCR, along with advanced next-generation sequencing (NGS) techniques, are highly effective in characterizing pathogens down to the genetic level. Typically, these techniques (NGS) are applied either to trace back outbreaks by identifying the pathogen and its possible source through phylogenetic analysis or for experimental or research purposes.

#### Description of the methodology

These methods require genetic material (Deoxyribonucleic acid (DNA) and ribonucleic acid (RNA)), which can be extracted from samples collected on animal farms using commercially available kits (87). The repertoire of techniques includes PCR, quantitative polymerase chain reaction (qPCR), reverse transcriptase PCR (RT-PCR), nucleic acid sequence-based amplification, as well as NGS techniques such as 16S rRNA gene sequencing and metagenomics (88).

#### Advantages

Molecular methods are considered as highly sensitive and specific, capable of detecting even very small quantities of genetic material to ensure accurate identification (89). These methods provide rapid results, which is especially valuable in outbreak investigations and in situations where culturing is difficult or impractical. NGS techniques offer comprehensive analysis, revealing detailed information about the genetic makeup of pathogens, including potential resistance genes and virulence factors. Their versatility allows application to a wide range of samples, including environmental, animal, and food samples, making them highly adaptable for various research and diagnostic purposes. Real-time PCR, in particular, facilitates the quantification of pathogen load, crucial for assessing infection severity and the effectiveness of C&D measures. The details on NGS techniques are beyond the scope of this review; however, readers are encouraged to refer to applied aspects of NGS for further information (90, 91).

#### Limitations

These methods can be costly, requiring expensive and specialized instrumentation and reagents, and trained personnel to perform and interpret the results (17). The complexity of these techniques can lead to technical errors and contamination. Additionally, these methods typically require high-quality genetic material, and the presence of inhibitors in the samples can interfere with the accuracy of the analysis. The extensive data generated by NGS can be challenging to analyze and interpret, requiring sophisticated bioinformatic tools and expertise.

However, a major limitation of this methodology, particularly the qPCR approach, is its inability to differentiate between nucleic acids from viable and non-viable organisms. This can lead to an overestimation of microbial contamination or infection risk, especially in post-disinfection assessments where non-viable cells may still be present (92). In a laboratory-based study by Buttner et al. (93), a comparative assessment was performed between traditional cultivation methods and qPCR across various surface substrates using a single target microorganism. The results demonstrated that cultivation methods detected only a small number of viable cells, whereas qPCR produced considerably higher measurements. Therefore, careful interpretation of threshold cycle values is needed to accurately interpret the relevance of qPCR results (24). Emerging techniques like the propidium monoazide qPCR method (PMA-qPCR) aim to address this, but routine on-farm application remains limited by cost and complexity (94).

#### Overall reflection

Molecular methods can serve as powerful tools for evaluating the quality of the C&D process, especially in detecting hard-to-culture or fastidious organisms that might evade detection through traditional methods. Molecular methods, particularly PCR and qPCR, are increasingly used in farm settings for detecting pathogens such as *Coxiella burnetii* and *Mycobacterium avium* subsp. *paratuberculosis* in environmental swabs or dust samples (95, 96). Furthermore, they can identify pathogens on a strain or even subtype level and offer significantly faster turnaround times compared to culture-based approaches, making them applicable for real-time monitoring of farm hygiene. However, due to the cost and effort involved, these methods are more practical for addressing persistent issues rather than for routine use.

## 4 Comparison of methods

Given the importance of hygiene measures within animal production systems, an effective method to evaluate hygiene should serve as more than just an indicator of the success of C&D procedures. It should also serve as a stringent control measure with proper identification of critical control points, validating the proper execution of every step of the process (56). An ideal assessment method for the evaluation of C&D should possess several critical attributes, including high sensitivity and specificity in detecting microorganisms, user-friendliness, rapidity, cost-effectiveness, consistent performance on both wet and dry surfaces, suitability for curved or rough surfaces, non-destructiveness, non-toxicity, resilience to chemical residues, strong repeatability and reproducibility. It should also provide recordable, and tamper-proof results, deliver objective and quantitative data, be suitable for real-time monitoring, and allow for trend analysis (22, 33, 60).

However, based on the analyzed studies in this review, there is no single method meeting all the above-mentioned criteria, with each method having both advantages and disadvantages concerning its use in evaluating C&D in a farm context.

Table 2 highlights the comparison of various methods for assessing the effectiveness of C&D procedures. In general, the convenient and most widespread method for assessments of C&D efficacy on farms is visual inspection, but visual inspection alone is considered as an unreliable indicator of cleaning effectiveness and is insufficient for accurately assessing the hygiene status (20, 97). Similarly, other kinds of visual examination such as UV fluorescence are subjective methods that are more applicable to the training of farmers. The ATP measurements can provide a more objective (quantitative) identification for use in critical or difficult-to-clean sampling points in comparison with the visual examination (20, 22). Additionally, total adenylate content, which consists of ATP, ADP, and AMP, can be used as a more reliable indicator of residual contamination in different conditions. At the same time, ATP bioluminescence measures overall cleanliness by detecting ATP in all living cells and organic matter, but it cannot distinguish harmful pathogens. In contrast, luciferase-based tests target specific viable pathogens with higher accuracy but are costlier and need specialized equipment, limiting their use in routine field inspections. The redox potential measurement technique to assess microbiological activity, which does not depend on surfaces, can be innovative applications for the agricultural sector. Yet it requires further technological development (75). One feasible approach could involve combining visual inspections with rapid tests to mitigate the added expenses associated with microbiological examinations (24).

**Table 2:**
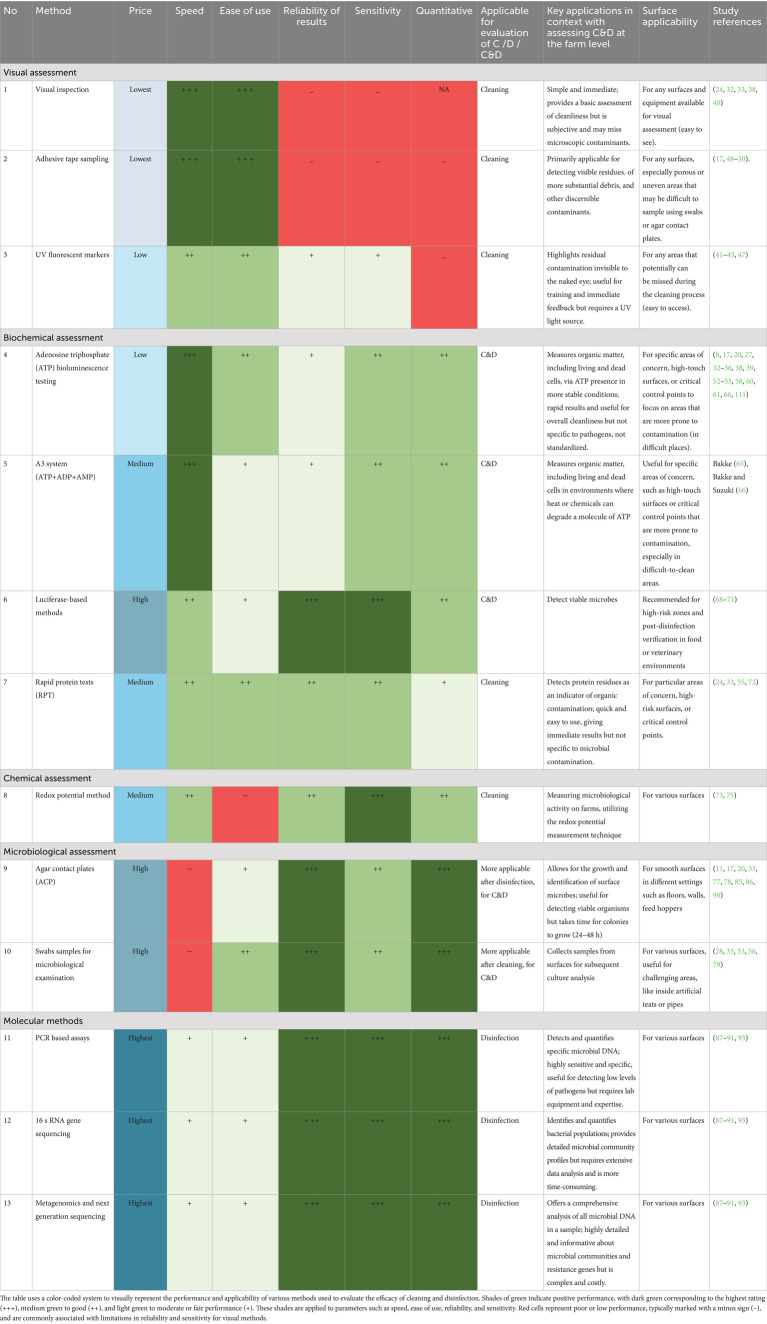
Comparison of methods for evaluating the efficacy of cleaning and disinfection (C&D) procedures.

However, a combination of visual examination and microbiological tests is more relevant in the case of a longer-term study/evaluation. For example, in pig production, according to Heinemann et al. (24), hygiene has already been proposed as a critical control point for on-farm assessment with daily visual inspections and additional monitoring of C&D procedures. One potential suggestion was to implement hygienogram scores similar to those already established in poultry farming, by investigating the TVC using agar plates to enhance the regular assessment of C&D practices (11). Introducing a system akin to hygienogram scores in piggery farm management could potentially enhance cleanliness but requires further development, particularly since the highest bacterial loads were detected at sampling points where ACP are not suitable for use (20, 98). Furthermore, microbiological tests could be used to target suspected pathogens in cases of persistent health issues and severe illnesses. The molecular methods can be applied during outbreak investigation, especially when the causative agent is unknown or to carry out evidence-based trace backing of contamination source(s). Moreover, the higher cost and complexity of molecular assays compared to traditional culture methods may restrict their routine application in livestock facilities.

It’s also critical to comprehend how each technique contributes to the detection of biofilms, which significantly reduce the effectiveness of C&D by shielding microorganisms from disinfectants and contributing to the development of antimicrobial resistance (15). Standard visual and ATP-based assessments fail to detect biofilm-embedded microbes. Culture-based methods may also underestimate contamination from biofilm-embedded microorganisms, and PCR may detect DNA from dead biofilm cells, complicating interpretation (93). Chemical methods testing and emerging biosensors (e.g., redox) provide improved sensitivity for early biofilm detection (65, 75). A multipronged strategy including mechanical removal, biofilm-active disinfectants, and enhanced monitoring is essential for robust biofilm control. However, the detailed discussion on biofilm detection and control strategies in livestock farming environments is beyond the scope of this review. Readers seeking in-depth insights are encouraged to consult recent comprehensive reviews focused specifically on biofilm management in agricultural and veterinary contexts (99–101).

Generally, it is important to select appropriate sampling methods based on the specific objectives and requirements of the assessment (29). The selection of testing methodologies should be an evidence-based process, aligning with key variables such as potential surface contamination, the specific hazards targeted by the C&D regimen, and the required level of cleanliness specific to each surface. Moreover, factors like when, where, and how to sample should be considered. Consequently, these considerations must guide decisions regarding the timing and methodology of sampling (29). Subjectivity and variation in individual perception may affect the precision of outcome. If a single individual is responsible for both the cleaning process and the visual inspection of farm facilities, there exists the possibility of overlooked areas during cleaning being similarly disregarded during the inspection (38, 55). Similarly, interpreting monitoring results is hindered by the absence of universally accepted guidelines defining when a surface is adequately cleaned (51).

Our analysis indicates that there are currently a limited number of studies describing the various techniques for evaluating hygiene in farm facilities and comparing their sensitivity and specificity under field conditions. Furthermore, research on the impact of different materials surfaces (wood, plastic, metal, concrete, etc.) on hygiene outcomes is limited, highlighting the need for further investigation in this area.

**Figure 7:**
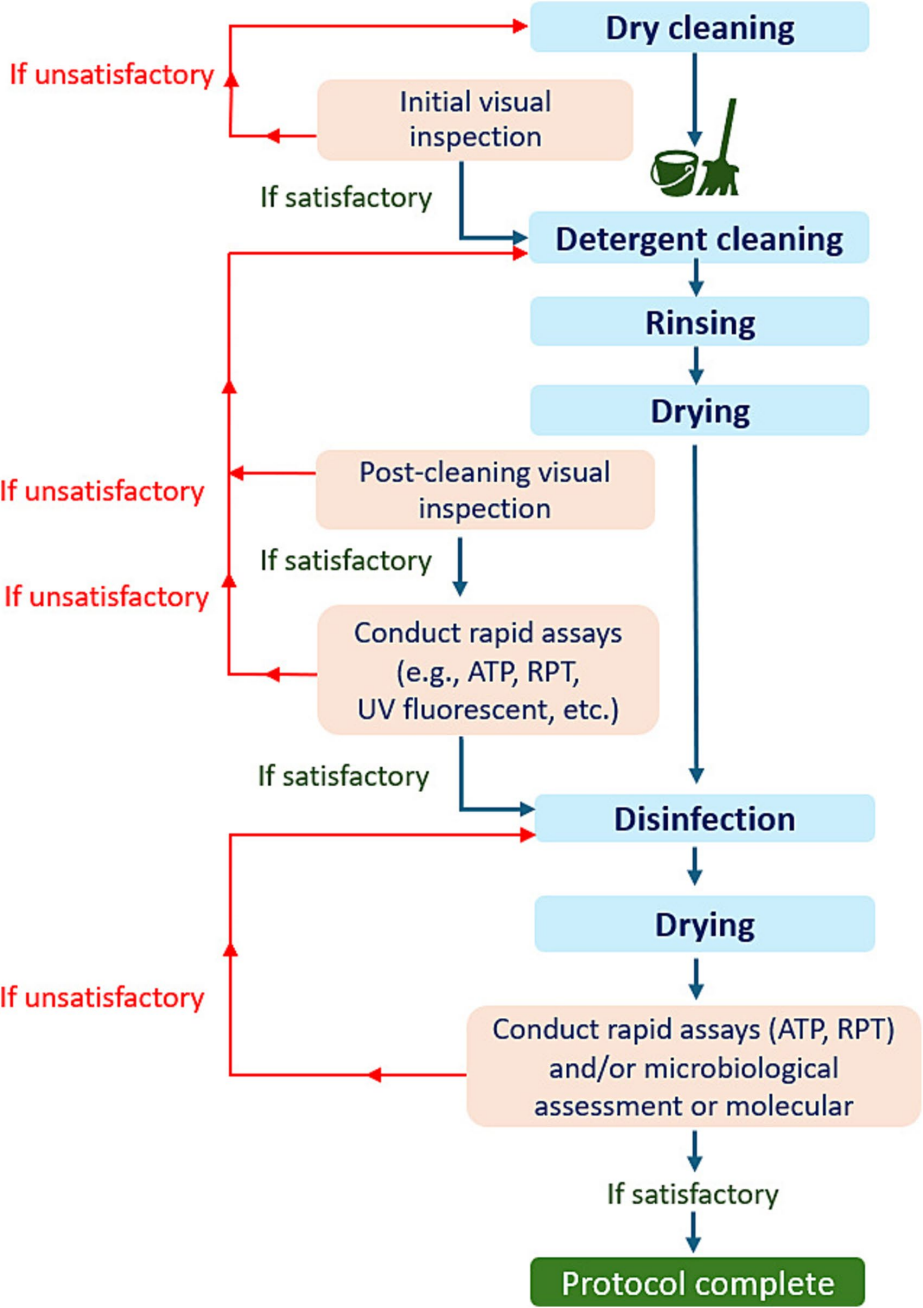
An illustration of the decision tree for the comprehensive evaluation protocol for C&D procedures.

Finally, the evaluation of hygiene effectiveness in diverse settings, particularly in the context of livestock farming, presents a complex challenge. To address this challenge effectively, it is imperative to integrate various assessment methods within a comprehensive protocol (Figure 7). This multi-step approach ensures that the C&D process is thoroughly assessed at each stage to maintain appropriate standards of hygiene. It is an ideal scenario which will probably not always be feasible under field condition.

As shown in Figure 7, the proper evaluation protocol should involve the following key steps:

(a) *Initial visual inspection*: A thorough visual examination should be conducted before cleaning begins.
(b) *Post-cleaning visual inspection*: After the cleaning and drying process, another visual inspection can be performed.

- *Decision point*: If notable organic residues or missed areas are detected during the visual examination, the detergent cleaning process should be repeated. If not, can proceed with rapid tests (such as ATP, or RPT).

(c) *Post-test evaluation*: If the results from rapid tests fail to meet established benchmarks, detergent cleaning should be repeated. If the results are satisfactory, proceed with disinfection.
(d) *Post-disinfection testing*: After disinfection and drying, samples should be tested using rapid tests, microbiological methods, or a combination of both. In the case of an outbreak, molecular testing can also be employed.

- *Final decision*: If the results of the applied tests fall below acceptable standards, the disinfection must be repeated.

Finally, to enhance hygiene management, it is highly beneficial to develop a farm-specific C&D protocol in collaboration with a supervising veterinarian, tailored to the farm’s needs, health status, and current epidemic conditions, as suggested by Heinemann et al. (24). This protocol would involve task verification similar to self-monitoring controls in the food industry. Regular in-house training sessions, possibly led by a specialized consultant and conducted periodically, could improve procedural efficiency and prevent lapses. Effective C&D also depend on the qualifications and understanding of risks by the personnel involved (13, 102, 103). Engaging professional cleaning contractors is advantageous, a practice commonly adopted in poultry production. Past studies have demonstrated that the efficacy of C&D performed by professional cleaning firms surpasses that conducted by in-house farm staff (11, 24). Therefore, it is essential to enhance awareness of the critical importance of hygiene in livestock production.

Furthermore, improving the evaluation approach and developing a comprehensive protocol should involve the integration of cutting-edge technologies, along with a forward-looking discussion on future advancements and trends in the field. Emerging technologies such as nanomaterial-based antimicrobials, photodynamic treatments, and pulsed light disinfection offer promising future alternatives to conventional disinfectants, particularly for targeting biofilm-associated pathogens (104–106). Additionally, intelligent sensors using electrochemical or optical biosensing, often integrated with artificial intelligence (AI) and Internet of Things (IoT) platforms, are being developed for real-time hygiene monitoring in food and farm environments (107, 108). These tools can detect residual ATP, pathogens, or environmental markers with high precision, enabling proactive sanitation management (41, 109). The integration of diverse technologies, such as biotechnology, physical technologies, and information technology, offers promising opportunities to enhance the evaluation of C&D effectiveness (92, 110, 111). Biotechnology enables the development of sensitive diagnostic tools and biosensors for rapid pathogen detection, while physical technologies can provide objective measurements of hygiene levels. Information technology, including AI, machine learning, and IoT systems, can further support real-time data collection, automated analysis, and decision-making. By combining these approaches, a more comprehensive, efficient, and data-driven framework for hygiene assessment can be established. This multidisciplinary integration not only improves accuracy and response time but also promotes the development of smart, scalable systems tailored for modern farm environments.

## 5 Limitations

This review has some limitations. First, it is a narrative review, while based on a systematic literature search, no quantitative synthesis or meta-analytic statistics were performed. Instead, the review offers a qualitative and comparative evaluation of methods used to assess C&D efficacy in reducing pathogen presence. Second, the scope was deliberately limited to farm livestock housing. Studies related to companion animals, laboratory animals, or other facility types were excluded to maintain a focused and practically relevant discussion. Finally, the review does not cover all existing C&D practices. Rather, it emphasizes the field applicability and comparative performance of selected field applicable evaluation methods, including biochemical, chemical, cultural, and molecular techniques. While this targeted approach enhances practical relevance, it may exclude broader methodological perspectives.

## 6 Conclusion

Assessing hygiene and microbial contamination in farm environments necessitates an evidence-based approach that leverages the strengths of multiple assessment methods. While no single method is flawless, the judicious combination of various techniques within a comprehensive C&D evaluation protocol provides a more holistic and accurate understanding of hygiene conditions. This integrated approach enables more precise monitoring and control of microbial contamination, ultimately enhancing the effectiveness of hygiene practices. The present study recommends choosing the appropriate combination of methods based on the specific needs of the farm, the frequency of evaluation, and the epidemiological status of the farm, district, and region. This strategic selection is crucial not only for improving hygiene management but also for ensuring the overall well-being of the animals and improving animal health. Also, periodic sensitization and training of the farm staff can improve the compliance and procedural efficiency. Future research should focus on developing comprehensive evaluation models for assessing C&D effectiveness, optimizing sampling strategies to improve accuracy and efficiency, and advancing automated and intelligent detection technologies. The integration of AI-driven systems, real-time biosensors, and IoT-based tools holds strong potential to enhance the reliability and responsiveness of hygiene monitoring in farm settings.

## References

[1] Jimenez CE Keestra S Tandon P Cumming O Pickering AJ Moodley A. Biosecurity and water, sanitation, and hygiene (WASH) interventions in animal agricultural settings for reducing infection burden, antibiotic use, and antibiotic resistance: a one health systematic review. Lancet Planetary Health. (2023) 7:e418–34. doi: 10.1016/S2542-5196(23)00049-9, PMID: 37164518

[2] Scollo A Perrucci A Stella M Ferrari P Robino P Nebbia P. Biosecurity and hygiene procedures in pig farms: effects of a tailor-made approach as monitored by environmental samples. Animals. (2023) 13:1262. doi: 10.3390/ani13071262, PMID: 37048519 PMC10093544

[3] Alarcón LV Allepuz A Mateu E. Biosecurity in pig farms: a review. Porcine Health Manag. (2021) 7:5. doi: 10.1186/s40813-020-00181-z 33397483 PMC7780598

[4] Alloui N Sellaoui SS Ayachi A Bennoune O. Evaluation of biosecurity practices in a laying hens farm using Biocheck.UGent. Multidiscip Sci J. (2021) 3:e2021014. doi: 10.29327/multiscience.2021014

[5] Wales AD Gosling RJ Bare HL Davies RH. Disinfectant testing for veterinary and agricultural applications: a review Zoonoses. Public Health. (2021) 68:361–75. doi: 10.1111/zph.12830, PMID: 33939312

[6] Militzer N McLaws M Rozstalnyy A Li Y Dhingra M Auplish A. Characterising biosecurity initiatives globally to support the development of a progressive management pathway for terrestrial animals: a scoping review. Animals. (2023) 13:2672. doi: 10.3390/ani13162672, PMID: 37627463 PMC10451226

[7] Alarcon P Marco-Jimenez F Horigan V Ortiz-Pelaez A Rajanayagam B Dryden A. A review of cleaning and disinfection guidelines and recommendations following an outbreak of classical scrapie. Prev Vet Med. (2021) 193:105388. doi: 10.1016/j.prevetmed.2021.105388 34098231

[8] Dewulf J Van Immerseel FV. Biosecurity in animal production and veterinary medicine. Acco: Leuven Publishers (2018).

[9] Gautam R Lahodny G Bani-Yaghoub M Morley PS Ivanek R. Understanding the role of cleaning in the control of *Salmonella Typhimurium* in grower-finisher pigs: a modelling approach. Epidemiol Infect. (2014) 142:1034–49. doi: 10.1017/S0950268813001805, PMID: 23920341 PMC9151178

[10] Gelaude P Schlepers M Verlinden M Laanen M Dewulf J. Biocheck.UGent: a quantitative tool to measure biosecurity at broiler farms and the relationship with technical performances and antimicrobial use. Poult Sci. (2014) 93:2740–51. doi: 10.3382/ps.2014-04002, PMID: 25193257

[11] Maertens H De Reu K Van Weyenberg S Van Coillie E Meyer E Van Meirhaeghe H. Evaluation of the hygienogram scores and related data obtained after cleaning and disinfection of poultry houses in Flanders during the period 2007 to 2014. Poult Sci. (2018) 97:620–7. doi: 10.3382/ps/pex327, PMID: 29211907

[12] Mannion C Leonard FC Lynch PB Egan J. Efficacy of cleaning and disinfection on pig farms in Ireland. Vet Rec. (2007) 161:371–5. doi: 10.1136/vr.161.11.371, PMID: 17873266

[13] Martelli F Lambert M Butt P Cheney T Tatone FA Callaby R. Evaluation of an enhanced cleaning and disinfection protocol in *Salmonella* contaminated pig holdings in the United Kingdom. PLoS One. (2017) 12:e0178897. doi: 10.1371/journal.pone.0178897, PMID: 28594930 PMC5464571

[14] Dhaka P Chantziaras I Vijay D Bedi JS Makovska I Biebaut E. Can improved farm biosecurity reduce the need for antimicrobials in food animals? A scoping review. Antibiotics. (2023) 12:893. doi: 10.3390/antibiotics12050893, PMID: 37237795 PMC10215210

[15] Richter AM Konrat K Osland AM Brook E Oastler C Vestby LK. Evaluation of biofilm cultivation models for efficacy testing of disinfectants against *Salmonella typhimurium* biofilms. Microorganisms. (2023) 11:761. doi: 10.3390/microorganisms11030761 36985334 PMC10052953

[16] Maillard JY Centeleghe I. How biofilm changes our understanding of cleaning and disinfection Antimicrob resist. Infect Control. (2023) 12:95. doi: 10.1186/s13756-023-01290-4, PMID: 37679831 PMC10483709

[17] Griffith C. Surface sampling and the detection of contamination In: J Holah, HLM Lelieveld, D Gabric (eds.) Handbook of Hygiene Control in the Food Industry 2nd Edition -June 17, 2016. Imprint: Woodhead Publishing (2016). 673–96.

[18] HanssonIDzieciolowskiTRydénJBoqvistS. Evaluation of cleaning and disinfection procedures on poultry farms. Poult Sci. (2025) 2025:105453. doi: 10.1016/j.psj.2025.10545340570462

[19] Walia K Argüello H Lynch H Grant J Leonard FC Lawlor PG. The efficacy of different cleaning and disinfection procedures to reduce *Salmonella* and *Enterobacteriaceae* in the lairage environment of a pig abattoir. Int J Food Microbiol. (2017) 246:71. doi: 10.1016/j.ijfoodmicro.2017.02.002 28189901

[20] Luyckx K Dewulf J Van Weyenberg S Herman L Zoons J Vervaet E. Comparison of sampling procedures and microbiological and non-microbiological parameters to evaluate cleaning and disinfection in broiler houses. Poult Sci. (2015) 94:740–9. doi: 10.3382/ps/pev019 25681611

[21] Hancox LR Le Bon M Dodd CER Mellits KH. Inclusion of detergent in a cleaning regime and effect on microbial load in livestock housing. Vet Rec. (2013) 173:167–7. doi: 10.1136/vr.101392, PMID: 23839725 PMC3756521

[22] Luyckx K Van Weyenberg S Dewulf J Herman L Zoons J Vervaet E. On-farm comparisons of different cleaning protocols in broiler houses. Poult Sci. (2015) 94:1986–93. doi: 10.3382/ps/pev143 26047671

[23] Makovska I Chantziaras I Caekebeke N Dhaka P Dewulf J. Assessment of cleaning and disinfection practices on pig farms across ten European countries. Animals. (2024) 14:593. doi: 10.3390/ani14040593, PMID: 38396561 PMC10886142

[24] Heinemann C Meyer I Bögel FT Schmid SM Hayer JJ Steinhoff-Wagner J. Individual training for farmers based on results from protein and ATP rapid tests and microbiological conventional cultural methods improves hygiene in pig fattening pens. J Anim Sci. (2019) 98:skz389. doi: 10.1093/jas/skz389 PMC6978896 31875908

[25] Pedersen L Houe H Rattenborg E Nielsen LR. Semi-quantitative biosecurity assessment framework targeting prevention of the introduction and establishment of *Salmonella Dublin* in dairy cattle herds. Animals. (2023) 13:2649. doi: 10.3390/ani13162649, PMID: 37627440 PMC10451256

[26] Ismaïl R Aviat F Michel V Le Bayon I Gay-Perret P Kutnik M. Methods for recovering microorganisms from solid surfaces used in the food industry: a review of the literature. Int J Environ Res Public Health. (2013) 10:6169–83. doi: 10.3390/ijerph10116169, PMID: 24240728 PMC3863893

[27] Lappalainen J Loikkanen S Havana M Karp M Sjöberg A-M Wirtanen G. Microbial testing methods for detection of residual cleaning agents and disinfectants—prevention of ATP bioluminescence measurement errors in the food industry. J Food Prot. (2000) 63:210–5. doi: 10.4315/0362-028X-63.2.210, PMID: 10678426

[28] Moore G Griffith C. Problems associated with traditional hygiene swabbing: the need for in-house standardization. J Appl Microbiol. (2007) 103:1090–103. doi: 10.1111/j.1365-2672.2007.03330.x, PMID: 17897214

[29] De Reu K. Luyckx K. Van Coillie E. Heyndrickx M. Dewulf J. Maes S.. (2019). Implications, efficiency and evaluation of cleaning and disinfection in commercial broiler farms. In Proceeding on LVI symposium Cientifico de Avicultura. pp. 33–39.

[30] Huang Y-S Chen Y-C Chen M-L Cheng A Hung I-C Wang J-T. Comparing visual inspection, aerobic colony counts, and adenosine triphosphate bioluminescence assay for evaluating surface cleanliness at a medical center. Am J Infect Control. (2015) 43:882–6. doi: 10.1016/j.ajic.2015.03.027, PMID: 25952617

[31] Rose N Mariani J Drouin P Toux JY Rose V Colin P. A decision-support system for *Salmonella* in broiler-chicken flocks. Prev Vet Med. (2003) 59. doi: 10.1016/S0167-5877(03)00056-4, PMID: 12719015

[32] Huneau-Salaün A Michel V Balaine L Petetin I Eono F Ecobichon F. Evaluation of common cleaning and disinfection programmes in battery cage and on-floor layer houses in France. Br Poult Sci. (2010) 51:204–12. doi: 10.1080/00071661003745794, PMID: 20461581

[33] Heinemann Leubner CD Hayer JJ Steinhoff-Wagner J. Hygiene management in newborn individually housed dairy calves focusing on housing and feeding practices. J Anim Sci. (2021) 99:skaa391. doi: 10.1093/jas/skaa391 33279999 PMC7799592

[34] Chancy A Santschi DE Paquet ÉR Renaud DL Gauthier ML Charbonneau É. Standardization and validation of ATP luminometry as a diagnostic tool to assess the cleanliness of feeding equipment in preweaning calves. J Dairy Sci. (2023) 106:6263–74. doi: 10.3168/jds.2023-23227, PMID: 37500439

[35] Lindell IC Lundh Å Sjaunja KS Cederholm M. Adenosine triphosphate bioluminescence for hygiene testing of rubber liners and tubes on dairy farms. J Dairy Sci. (2018) 101:2438–47. doi: 10.3168/jds.2017-13466, PMID: 29290436

[36] Renaud DL Kelton DF LeBlanc SJ Haley DB Jalbert AB Duffield TF. Validation of commercial luminometry swabs for total bacteria and coliform counts in colostrum-feeding equipment J. Dairy Sci. (2017) 100:9459–65. doi: 10.3168/jds.2017-13228 28918141

[37] Potter T. Calf rearing hygiene – from on-farm assessment to client engagement. Livestock. (2025) 30:49–54. doi: 10.12968/live.2024.0038

[38] Van Driessche L Santschi DE Paquet É Renaud D Charbonneau É Gauthier M-L. Hygiene management practices and adenosine triphosphate luminometry of feeding equipment in preweaning calves on dairy farms in Quebec, Canada. J Dairy Sci. (2023) 106:8885–96. doi: 10.3168/jds.2023-23626, PMID: 37641362

[39] Alvarado A Cabahug J. ATP bioluminescence method as a rapid tool for assessment of cleanliness of commercial animal transport trailers. Can Biosyst Eng. (2022) 62:1. doi: 10.7451/CBE.2020.62.5.1

[40] Chen YC Huang HM Lin PY Shi ZY. Comparing visual inspection and performance observation for evaluation of hospital cleanliness. Am J Infect Control. (2021) 49:1511–4. doi: 10.1016/j.ajic.2021.07.011, PMID: 34314756

[41] Gorji HT Van Kessel JAS Haley BJ Husarik K Sonnier J Shahabi SM. Deep learning and multiwavelength fluorescence imaging for cleanliness assessment and disinfection in food services. Front Sens. (2022) 3:977770. doi: 10.3389/fsens.2022.977770

[42] Weese JS Lowe T Walker M. Use of fluorescent tagging for assessment of environmental cleaning and disinfection in a veterinary hospital. Vet Rec. (2012) 171:217–7. doi: 10.1136/vr.100796, PMID: 22798345

[43] Carling PC Bartley JM. Evaluating hygienic cleaning in health care settings: what you do not know can harm your patients. Am J Infect Control. (2010) 38:S41–50. doi: 10.1016/j.ajic.2010.03.004, PMID: 20569855

[44] Aouthmany S Mehalik H Bailey M Pei M Syed S Brickman K. Use of ultraviolet light in graduate medical education to assess confidence among residents and fellows in handwashing instruction. Antimicrobial Stewardship and Healthcare Epidemiology. (2022) 2:e65. doi: 10.1017/ash.2021.208, PMID: 36483347 PMC9726597

[45] Szilágyi L Haidegger T Lehotsky Á Nagy M Csonka E-A Sun X. A large-scale assessment of hand hygiene quality and the effectiveness of the “WHO 6-steps”. BMC Infect Dis. (2013) 13:249. doi: 10.1186/1471-2334-13-249 23718728 PMC3689096

[46] Harrison O Dahmer P Gebhardt J Paulk C Woodworth J Jones C. Evaluation of biosecurity measures on a swine operation using Glo germ powder as a visible learning aid. J Swine Health Prod. (2022) 30:362–6. doi: 10.54846/jshap/1289

[47] Assadian O Harbarth S Vos M Knobloch JK Asensio A Widmer AF. Practical recommendations for routine cleaning and disinfection procedures in healthcare institutions: a narrative review. J Hosp Infect. (2021) 113:104–14. doi: 10.1016/j.jhin.2021.03.010, PMID: 33744383

[48] Bisha B Brehm-Stecher BF. Simple adhesive-tape-based sampling of tomato surfaces combined with rapid fluorescence in situ hybridization for *Salmonella* detection. Appl Environ Microbiol. (2009) 75:1450–5. doi: 10.1128/AEM.01944-08, PMID: 19124588 PMC2648154

[49] Deshpande A Donskey CJ. Practical approaches for assessment of daily and post-discharge room disinfection in healthcare facilities. Curr Infect Dis Rep. (2017) 19:32. doi: 10.1007/s11908-017-0585-6 28770497

[50] Meider J Messal C. Faster evaluation of contaminated surfaces for mould inspections by tape sampling. J Biomed Res Environ Sci. (2021) 516-522. doi: 10.37871/jbres1268

[51] Moazzami M Bergenkvist E Boqvist S Frosth S Langsrud S Møretrø T. Assessment of ATP-bioluminescence and dipslide sampling to determine the efficacy of slaughterhouse cleaning and disinfection compared with total aerobic and Enterobacterales counts. J Food Prot. (2023) 86:100155. doi: 10.1016/j.jfp.2023.100155, PMID: 37659478

[52] Buczinski S Morin M-P Roy J-P Rousseau M Villettaz-Robichaud M Dubuc J. Use of ATP luminometry to assess the cleanliness of equipment used to collect and feed colostrum on dairy farms. J Dairy Sci. (2022) 105:1638–48. doi: 10.3168/jds.2021-21023, PMID: 34802728

[53] Vilar MJ Rodríguez-Otero JL Diéguez FJ Sanjuán ML Yus E. Application of ATP bioluminescence for evaluation of surface cleanliness of milking equipment. Int J Food Microbiol. (2008) 125:357–61. doi: 10.1016/j.ijfoodmicro.2008.04.024, PMID: 18547666

[54] Bautista DA Sprung DW Barbut S Griffiths MW. A sampling regime based on an ATP bioluminescence assay to assess the quality of poultry carcasses at critical control points during processing. Food Res Int. (1997) 30:803–9. doi: 10.1016/S0963-9969(98)00049-0

[55] Yi SW Cho A Kim E Oh SI Roh JH Jung YH. Evaluation of adenosine triphosphate testing for on-farm cleanliness monitoring compared to microbiological testing in an empty pig farrowing unit. J Anim Sci Technol (Seoul). (2020) 62:682–91. doi: 10.5187/jast.2020.62.5.682, PMID: 33089233 PMC7553838

[56] Mateus-Vargas RH Butenholz K Volkmann N Sürie C Kemper N Schulz J. Boot swabs to evaluate cleaning and disinfection success in poultry barns. Agriculture. (2022) 12:57. doi: 10.3390/agriculture12010057

[57] Nante N Ceriale E Messina G Manzi P. Effectiveness of ATP bioluminescence to assess hospital cleaning: a review. Eur J Pub Health. (2017) 24. doi: 10.1093/eurpub/cku163.030 PMC5584088 28900359

[58] Letsch FG Welch MW Meyer M Hedblom GA Parr E Classen DM. Evaluation of ATP bioluminescence for rapid determination of cleanliness of livestock trailers after a commercial wash. Transl Anim Sci. (2024) 8:txae052. doi: 10.1093/tas/txae052 38651119 PMC11034431

[59] Green TA Russell SM Fletcher DL. Effect of chemical sanitizing agents on ATP bioluminescence measurements. J Food Prot. (1998) 61:1013–7. doi: 10.4315/0362-028X-61.8.1013, PMID: 9713763

[60] Turner DE Daugherity EK Altier C Maurer KJ. Efficacy and limitations of an ATP-based monitoring system. J Am Assoc Lab Anim Sci. (2010) 49:190–5. PMID: 20353694 PMC2846007

[61] Tetro J. A. Sattar S. A. (2021). The application of ATP bioluminescence for rapid monitoring of microbiological contamination on environmental surfaces: a critical review. InfectionControl tips. Available online at: https://infectioncontrol.tips/wp/wp-content/uploads/2021/11/ATP-Critical-Review.pdf [Accessed February 3, 2025].

[62] Aycicek H Oguz U Karci K. Comparison of results of ATP bioluminescence and traditional hygiene swabbing methods for the determination of surface cleanliness at a hospital kitchen. Int J Hyg Environ Health. (2006) 209:203–6. doi: 10.1016/j.ijheh.2005.09.007, PMID: 16503304

[63] Öz P Özgen Arun Ö. Evaluating the performance of ATP bioluminescence method by comparison with classical cultural method. Food Health. (2019):77–82. doi: 10.3153/FH19008

[64] Pistelok F Pohl A Stuczyński T Wiera B. Using ATP tests for assessment of hygiene risks. Ecol Chem Eng S. (2016) 23:259–70. doi: 10.1515/eces-2016-0018

[65] Bakke M. A comprehensive analysis of ATP tests: practical use and recent progress in the total adenylate test for the effective monitoring of hygiene. J Food Prot. (2022) 85:1079–95. doi: 10.4315/JFP-21-384, PMID: 35503956

[66] Bakke M Suzuki S. Development of a novel hygiene monitoring system based on the detection of total adenylate (ATP+ADP+AMP). J Food Prot. (2018) 81:729–37. doi: 10.4315/0362-028X.JFP-17-432, PMID: 29611732

[67] Denis-Robichaud J Barbeau-Grégoire N Gauthier M-L Dufour S Roy J-P Buczinski S. Validity of luminometry and bacteriological tests for diagnosing intramammary infection at dry-off in dairy cows. J Dairy Sci. (2024) 107:7221–9. doi: 10.3168/jds.2024-24693, PMID: 38788849

[68] Kurtboke I. Bacteriophages. London: IntechOpen (2012).

[69] Wei S Chelliah R Rubab M Oh D-H Uddin MJ Ahn J. Bacteriophages as potential tools for detection and control of *Salmonella* spp. in food systems. Microorganisms. (2019) 7:11 570. doi: 10.3390/microorganisms7110570 31744260 PMC6920764

[70] Tanner NA Zhang Y Evans TC. Visual detection of isothermal nucleic acid amplification using pH-sensitive dyes. BioTechniques. (2015) 58:59–68. doi: 10.2144/000114253, PMID: 25652028

[71] Bai J Kim Y-T Ryu S Lee J-H. Biocontrol and rapid detection of food-borne pathogens using bacteriophages and Endolysins. Front Microbiol. (2016) 7:474. doi: 10.3389/fmicb.2016.00474, PMID: 27092128 PMC4824769

[72] Casini B Tuvo B Totaro M Aquino F Baggiani A Privitera G. Evaluation of the cleaning procedure efficacy in prevention of nosocomial infections in healthcare facilities using cultural method associated with high sensitivity luminometer for ATP detection. Pathogens. (2018) 7:71. doi: 10.3390/pathogens7030071, PMID: 30200291 PMC6161163

[73] Reichart O Szakmár K Jozwiak Á Felföldi J Baranyai L. Redox potential measurement as a rapid method for microbiological testing and its validation for coliform determination. Int J Food Microbiol. (2007) 114 2:143 148. doi: 10.1016/j.ijfoodmicro.2006.08.016 17229481

[74] Zhang D Wang Y Sun X Liu Y Zhou Y Shin H. Voltammetric, spectroscopic, and cellular characterization of redox functionality of eckol and phlorofucofuroeckol-a: a comparative study. J Food Biochem. (2019) 43:7. doi: 10.1111/jfbc.12845 31353689

[75] Yakdhane E Tőzsér D Haykir O Yakdhane A Labidi S Kiskó G. Recognition of environmental contaminant and pathogenic bacteria by means of redox potential methodology. MethodsX. (2024) 13:102811. doi: 10.1016/j.mex.2024.102811, PMID: 39022177 PMC11253682

[76] Erdősi O Szakmár K Reichart O Székely-Körmöczy P Laczay P. Application of the redox potential measurementbased rapid method in the microbial hygienic control. Acta Aliment. (2012) 41:45–55. doi: 10.1556/AAlim.2011.0005

[77] Ward PJ Fasenko GM Gibson S McMullen LM. A microbiological assessment of on-farm food safety cleaning methods in broiler barns. J Appl Poult Res. (2006) 15 2:326 332. doi: 10.1093/japr/15.2.326

[78] De Castro Burbarelli MF Polycarpo G DV Deliberali Lelis K Granghelli CA Carão De Pinho AC Ribeiro Almeida Queiroz S. Cleaning and disinfection programs against *Campylobacter jejuni* for broiler chickens: productive performance, microbiological assessment and characterization. Poult Sci. (2017) 96:3188–98. doi: 10.3382/ps/pex153, PMID: 28854757 PMC5850738

[79] Berghaus RD Thayer SG Law BF Mild RM Hofacre CL Singer RS. Enumeration of Salmonella and Campylobacter spp. in environmental farm samples and processing plant carcass rinses from commercial broiler chicken flocks. Appl Environ Microbiol. (2013) 79:4106–14. doi: 10.1128/AEM.00836-13, PMID: 23624481 PMC3697552

[80] Van Tassell JA Martin NH Murphy SC Wiedmann M Boor KJ Ivy RA. Evaluation of various selective media for the detection of *Pseudomonas* species in pasteurized milk. J Dairy Sci. (2012) 95 3:1568–74. doi: 10.3168/jds.2011-4958 22365238

[81] Hervert CJ Alles AS Martin NH Boor KJ Wiedmann M. Evaluation of different methods to detect microbial hygiene indicators relevant in the dairy industry. J Dairy Sci. (2016) 99:7033–42. doi: 10.3168/jds.2016-11074, PMID: 27394938

[82] Kawanishi T Shiraishi T Okano Y Sugawara K Hashimoto M Maejima K. New detection systems of bacteria using highly selective media designed by SMART: selective medium-design algorithm restricted by two constraints. PLoS One. (2011) 6:e16512. doi: 10.1371/journal.pone.0016512, PMID: 21304596 PMC3029383

[83] Prasad M Shetty SK Nair BG Pal S Madhavan A. A novel and improved selective media for the isolation and enumeration of *Klebsiella* species. Appl Microbiol Biotechnol. (2022) 106:8273–84. doi: 10.1007/s00253-022-12270-w, PMID: 36380193 PMC9726774

[84] Evangelopoulou G Burriel AR Solomakos N. Distinctive culture expressions of *Enterobacteria* interfering with isolation of *Salmonella* spp. during the application of the recommended ISO 6579-1:2017. Appl Sci. (2024) 14:953. doi: 10.3390/app14030953

[85] Dewaele I Ducatelle R Herman L Heyndrickx M De Reu K. Sensitivity to disinfection of bacterial indicator organisms for monitoring the *Salmonella Enteritidis* status of layer farms after cleaning and disinfection. Poult Sci. (2011) 90:1185–90. doi: 10.3382/ps.2010-01178, PMID: 21597057

[86] Tortorello ML. Indicator organisms for safety and quality—uses and methods for detection: Minireview. J AOAC Int. (2003) 86:1208–17. doi: 10.1093/jaoac/86.6.1208, PMID: 14979704

[87] Mirabile A Sangiorgio G Bonacci PG Bivona D Nicitra E Bonomo C. Advancing pathogen identification: the role of digital PCR in enhancing diagnostic power in different settings. Diagnostics. (2024) 14:1598. doi: 10.3390/diagnostics14151598, PMID: 39125474 PMC11311727

[88] Afonso CL Afonso AM. Next-generation sequencing for the detection of microbial agents in avian clinical samples. Vet Sci. (2023) 10:690. doi: 10.3390/vetsci10120690, PMID: 38133241 PMC10747646

[89] Wirtanen G. Salo S. (2004). DairyNET: hygiene control in Nordic dairies. VTT.

[90] Suminda GGD Bhandari S Won Y Goutam U Kanth PK Son Y-O. High-throughput sequencing technologies in the detection of livestock pathogens, diagnosis, and zoonotic surveillance Comput Struct. Biotechnol J. (2022) 20:5378–92. doi: 10.1016/j.csbj.2022.09.028, PMID: 36212529 PMC9526013

[91] Wensel CR Pluznick JL Salzberg SL Sears CL. Next-generation sequencing: insights to advance clinical investigations of the microbiome. J Clin Invest. (2022) 132:e154944. doi: 10.1172/JCI154944, PMID: 35362479 PMC8970668

[92] Ren M Yu X Mujumdar AS Yagoub AE-GA Chen L Zhou C. Visualizing the knowledge domain of pulsed light technology in the food field: a scientometrics review. Innov Food Sci Emerg Technol. (2021) 74:102823. doi: 10.1016/j.ifset.2021.102823

[93] Buttner MP Cruz P Stetzenbach LD Cronin T. Evaluation of two surface sampling methods for detection of *Erwinia herbicola* on a variety of materials by culture and quantitative PCR. Appl Environ Microbiol. (2007) 73:3505–10. doi: 10.1128/AEM.01825-06, PMID: 17416685 PMC1932667

[94] Liu Y Huang S Zhou J Zhang C Hu F Xiao Y. A new method for the rapid detection of the antibacterial and bacteriostatic activity of disinfectants based on Propidium Monoazide combined with real-time PCR. Front Microbiol. (2022) 13:1051162. doi: 10.3389/fmicb.2022.1051162 36425040 PMC9678941

[95] Fernández-Carrillo JDO-M Sellek RE Ortega-García MV Cabria-Ramos JC Bassy O. Development of a specific real-time PCR assay for simultaneous detection and differentiation of *Coxiella burnetii* strains from environmental soil samples. Lett Appl Microbiol. (2023) 76:ovad030. doi: 10.1093/lambio/ovad030 36841234

[96] Field NL Mee JF McAloon CG. Evaluation of environmental sampling for detection of *Mycobacterium avium* subspecies paratuberculosis in the pre-weaned calf area and calving area of infected dairy farms enrolled in a voluntary Johne’s disease control Programme. Animals. (2023) 13:669. doi: 10.3390/ani13040669, PMID: 36830456 PMC9951948

[97] Mustafa EA Babeker HA. Evaluation of the efficacy of cleaning and disinfection in broiler farms between rest periods in Khartoum state, Sudan. World J Pharm Pharmaceut Sci. (2018) 7:1893. doi: 10.20959/wjpps20187-11893

[98] Luyckx VCE Dewulf J Van Weyenberg S Herman L Zoons J Vervaet E. Identification and biocide susceptibility of dominant bacteria after cleaning and disinfection of broiler houses. Poult Sci. (2017) 96:938–49. doi: 10.3382/ps/pew355, PMID: 28158762

[99] Araújo D Silva AR Fernandes R Serra P Barros MM Campos AM. Emerging approaches for mitigating biofilm-formation-associated infections in farm, wild, and companion animals. Pathogens. (2024) 13:320. doi: 10.3390/pathogens13040320, PMID: 38668275 PMC11054384

[100] Bridier A Briandet R Thomas V Dubois-Brissonnet F. Resistance of bacterial biofilms to disinfectants: a review. Biofouling. (2011) 27:1017–32. doi: 10.1080/08927014.2011.626899, PMID: 22011093

[101] Su Y Yrastorza JT Matis M Cusick J Zhao S Wang G. Biofilms: formation, research models, potential targets, and methods for prevention and treatment. Adv Sci. (2022) 9:2203291. doi: 10.1002/advs.202203291 PMC9561771 36031384

[102] Carrique-Mas JJ Marín C Breslin M McLaren I Davies R. A comparison of the efficacy of cleaning and disinfection methods in eliminating *Salmonella* spp. from commercial egg laying houses. Avian Pathol. (2009) 38:419–24. doi: 10.1080/03079450903193768, PMID: 19937529

[103] Gosling R. A review of cleaning and disinfection studies in farming environments. Livestock. (2018) 23:232–7. doi: 10.12968/live.2018.23.5.232

[104] Eleftheriadou M Pyrgiotakis G Demokritou P. Nanotechnology to the rescue: using nano-enabled approaches in microbiological food safety and quality. Curr Opin Biotechnol. (2017) 44:87–93. doi: 10.1016/j.copbio.2016.11.012, PMID: 27992831 PMC5385268

[105] Gong C Li Y Gao R Xiao F Zhou X Wang H. Preservation of sturgeon using a photodynamic non-thermal disinfection technology mediated by curcumin. Food Biosci. (2020) 36:100594. doi: 10.1016/j.fbio.2020.100594

[106] Mohammad ZH Ahmad F Ibrahim SA Zaidi S. Application of nanotechnology in different aspects of the food industry. Discov Food. (2022) 2:12. doi: 10.1007/s44187-022-00013-9

[107] Dodero A Escher A Bertucci S Castellano M Lova P. Intelligent packaging for real-time monitoring of food-quality: current and future developments. Appl Sci. (2021) 11:3532. doi: 10.3390/app11083532

[108] Jiang Y Tran TH Collins M Williams L. Development of internet of things and artificial intelligence for intelligent sanitation systems: a literature review. J Infrastruct Policy Dev. (2024) 8:7889. doi: 10.24294/jipd.v8i12.7889

[109] Alenyorege EA Ma H Aheto JH Ayim I Chikari F Osae R. Response surface methodology centred optimization of mono-frequency ultrasound reduction of bacteria in fresh-cut Chinese cabbage and its effect on quality. LWT. (2020) 122:108991. doi: 10.1016/j.lwt.2019.108991

[110] Zhang H Mahunu GK Castoria R Apaliya MT Yang Q. Augmentation of biocontrol agents with physical methods against postharvest diseases of fruits and vegetables. Trends Food Sci Technol. (2017) 69:36–45. doi: 10.1016/j.tifs.2017.08.020

[111] Osimani A Garofalo C Clementi F Tavoletti S Aquilanti L. Bioluminescence ATP monitoring for the routine assessment of food contact surface cleanliness in a university canteen. Int J Environ Res Public Health. (2014) 11:10824–37. doi: 10.3390/ijerph111010824, PMID: 25329534 PMC4211008

